# Influence of Dietary Long-Chain Polyunsaturated Fatty Acids and ω6 to ω3 Ratios on Head Kidney Lipid Composition and Expression of Fatty Acid and Eicosanoid Metabolism Genes in Atlantic Salmon (*Salmo salar*)

**DOI:** 10.3389/fmolb.2020.602587

**Published:** 2020-12-14

**Authors:** Tomer Katan, Xi Xue, Albert Caballero-Solares, Richard G. Taylor, Matthew L. Rise, Christopher C. Parrish

**Affiliations:** ^1^Department of Ocean Sciences, Memorial University of Newfoundland, St. John's, NL, Canada; ^2^Cargill Animal Nutrition, Elk River, MN, United States

**Keywords:** salmon aquaculture, omega-6/omega-3 ratio, EPA+DHA, lipid metabolism, head kidney transcript expression, nutrigenomics, metabolomics, fatty acids

## Abstract

The interaction of dietary eicosapentaenoic acid and docosahexaenoic acid (EPA+DHA) levels with omega-6 to omega-3 ratios (ω6:ω3), and their impact on head kidney lipid metabolism in farmed fish, are not fully elucidated. We investigated the influence of five plant-based diets (12-week exposure) with varying EPA+DHA levels (0.3, 1.0, or 1.4%) and ω6:ω3 (high ω6, high ω3, or balanced) on tissue lipid composition, and transcript expression of genes involved in fatty acid and eicosanoid metabolism in Atlantic salmon head kidney. Tissue fatty acid composition was reflective of the diet with respect to C_18_ PUFA and MUFA levels (% of total FA), and ω6:ω3 (0.5–1.5). Fish fed 0.3% EPA+DHA with high ω6 (0.3% EPA+DHA↑ω6) had the highest increase in proportions (1.7–2.3-fold) and in concentrations (1.4-1.8-fold) of arachidonic acid (ARA). EPA showed the greatest decrease in proportion and in concentration (by ~½) in the 0.3% EPA+DHA↑ω6 fed fish compared to the other treatments. However, no differences were observed in EPA proportions among salmon fed the high ω3 (0.3 and 1.0% EPA+DHA) and balanced (1.4% EPA+DHA) diets, and DHA proportions were similar among all treatments. Further, the transcript expression of *elovl5a* was lowest in the 0.3% EPA+DHA↑ω6 fed fish, and correlated positively with 20:3ω3, 20:4ω3 and EPA:ARA in the head kidney. This indicates that high dietary 18:3ω3 promoted the synthesis of ω3 LC-PUFA. Dietary EPA+DHA levels had a positive impact on *elovl5a, fadsd5* and *srebp1* expression, and these transcripts positively correlated with tissue ΣMUFA. This supported the hypothesis that LC-PUFA synthesis is positively influenced by tissue MUFA levels in Atlantic salmon. The expression of *pparaa* was higher in the 0.3% EPA+DHA↑ω6 compared to the 0.3% EPA+DHA↑ω3 fed fish. Finally, significant correlations between head kidney fatty acid composition and the expression of eicosanoid synthesis-related transcripts (i.e., *5loxa, 5loxb, cox1, cox2, ptges2, ptges3*, and *pgds*) illustrated the constitutive relationships among fatty acids and eicosanoid metabolism in salmon.

## Introduction

Long-chain polyunsaturated fatty acids (LC-PUFA) such as eicosapentaenoic (EPA, 20:5ω3), docosahexaenoic (DHA, 22:6ω3) and arachidonic (ARA, 20:4ω6) acids play important roles in fish growth, metabolism (Sargent et al., 2002), neural development, health and reproduction (Sargent et al., 1999; Tocher, 2010, 2015). They are also important structural components of cell membranes (Calder, 2013), and act as precursors to eicosanoid metabolites such as prostaglandins, leukotrienes, thromboxanes, docosanoids, maresins and resolvins which regulate inflammatory and immune response processes (Calder, 2007, 2013; Arts and Kohler, 2009; Martinez-Rubio et al., 2013; Holen et al., 2015; Montero et al., 2019). The levels of EPA and DHA in fish feeds showed a large reduction (from ~ 20 to 5% of dietary lipid) over the past ~3 decades (Einen and Thomassen, 1998; Ytrestøyl et al., 2015; Sprague et al., 2016), due to decreasing global availability and rising market price of their fish oil (FO) sources (Turchini et al., 2009; Tacon and Metian, 2015; Froehlich et al., 2018). The reduction in FO and fish meal (FM), and the concomitant rise in incorporation of plant-based ingredients have influenced fish health (e.g., liver steatosis, altered phagocytic activity, and modulation of immune-related transcript expression) (Montero et al., 2003; Ruyter et al., 2006; Caballero-Solares et al., 2017), and their nutritional quality for human consumers (Sprague et al., 2016; De Roos et al., 2017).

Salmonids have a capacity to desaturate and elongate the precursors 18:3ω3 and 18:2ω6 to LC-PUFA metabolites (Tocher et al., 2000; Hixson et al., 2014; Katan et al., 2019). The enzymes involved in the bioconversion of both ω3 and ω6 pathways are the fatty acyl desaturases (FADS2D5, FADS2D6a, FADS2D6b and FADS2D6c: Zheng et al., 2005; Monroig et al., 2010) and fatty acyl elongases (ELOVL5a, ELOVL5b, ELOVL2, ELOVL4a and ELOVL4b: Hastings et al., 2004; Morais et al., 2009; Carmona-Antoñanzas et al., 2011; Zhao et al., 2019). Hepatic LC-PUFA synthesis in Atlantic salmon (*Salmo salar*) is influenced by precursor (18:2ω6 and/or 18:3ω3) availability, as well as the concentration of LC-PUFA (i.e., EPA, ARA, DHA) (Jordal et al., 2005; Glencross et al., 2015; Katan et al., 2019). This pathway is regulated at the transcriptional level and is mediated by transcription factors [e.g., liver X receptor (LXR), sterol regulatory element binding protein (SREBP) 1 and 2, and peroxisome proliferator-activated receptor (PPAR) α, β and γ (Carmona-Antoñanzas et al., 2014; Hixson et al., 2017; Katan et al., 2019; Emam et al., 2020)]. However, the constitutive regulation of this pathway in salmon head kidney is less well-understood (Betancor et al., 2014), and requires further investigation given the central role of this organ in haematopoetic and immune processes (Tort et al., 2003; Zapata et al., 2006; Gjøen et al., 2007).

Plant oils used in aquafeeds generally contain high levels of ω6 FA, and as such, these oils and farmed fish consuming them may provide inadequate ratios of ω6 to ω3 (ω6:ω3) fatty acids for the consumer (Pickova and Mørkøre, 2007; Weaver et al., 2008; Young, 2009). Further, ω6:ω3 plays an important role in fish immune response [through their conversion into pro- and anti-inflammatory eicosanoids (Furne et al., 2013; Holen et al., 2018)] and FA metabolism [e.g., impacting LC-PUFA synthesis and transcript expression of genes involved in this pathway (Torstensen and Tocher, 2010; Vagner and Santigosa, 2011; Katan et al., 2019)]. Previous studies revealed that diets with varying ω6:ω3 ratio (i.e., ~0.3–4.0) influenced the transcription of inflammation- and FA metabolism-related genes in Atlantic salmon head kidney (Martinez-Rubio et al., 2013; Holen et al., 2018). However, the interaction of dietary ω6:ω3 with EPA+DHA levels, and their nutritional impacts on eicosanoid and fatty acid metabolism in salmon, have not been fully elucidated.

Emam et al. (2020) recently showed that diets with different combinations of ω6:ω3 (high ω6, high ω3, or balanced) and EPA+DHA levels (0.3, 1.0, or 1.4%) affected muscle lipid composition, and the hepatic transcript expression of fatty acid synthesis-related genes in Atlantic salmon. The current study used the same diets and fish population as in Emam et al. (2020), with the aim to investigate how diets with low FO inclusion levels impact head kidney lipid composition, and the transcript expression of genes involved in fatty acid and eicosanoid metabolism. Furthermore, correlation analyses were used to relate head kidney lipid composition with transcript expression.

## Materials and Methods

### Experimental Diets and Animals

Five experimental diets were manufactured by Cargill Canada (formerly EWOS Canada; Surrey, BC, Canada), top-coated with different oil mixes at the Chute Animal Nutrition Centre (Dalhousie University, Truro, NS, Canada), and stored at −20°C until needed. Experimental diets contained different levels of FO, poultry fat, and vegetable oils (VO; i.e., soy oil, linseed oil, and rapeseed oil) to generate three levels of EPA+DHA (0.3, 1.0, and 1.4%, as formulated) and contrasting ω6:ω3 ratios (high ω6, high ω3, and balanced). The diets were as follows: 0.3% EPA+DHA with higher ω6 (0.3% EPA+DHA↑ω6), 0.3% EPA+DHA with higher ω3 (0.3% EPA+DHA↑ω3), 1.0% EPA+DHA with higher ω6 (1%EPA+DHA↑ω6), 1.0%EPA+DHA with higher ω3 (1%EPA+DHA↑ω3), and 1.4%EPA+DHA with a more balanced ω6:ω3 ratio (1.4% EPA+DHA/balanced). All diets were formulated to be isonitrogenous and isoenergetic and to meet the nutritional requirements of salmonids (National Research Council (NRC), 2011). Dietary formulations and their lipid compositions were published previously (Emam et al., 2020). However, as they pertain to the current study, they are included as Supplementary Material here (Supplementary Tables 1, 2).

Atlantic salmon smolts were transported from a regional salmon farm to the Dr. Joe Brown Aquatic Research Building (Ocean Sciences Centre, Memorial University of Newfoundland, Canada; October 2016). After their arrival, fish were graded by size in order to select the most uniform population, PIT (Passive Integrated Transponder; Easy AV, Avid Identification Systems, Norco, CA, USA)-tagged for individual identification, and kept in 3,800-litre tanks until the beginning of the feeding trial. Then, salmon post-smolts [210 ± 44 g mean initial weight ± standard deviation (SD)] were randomly distributed into twenty 620-litre tanks (40-41 fish tank^−1^), and subjected to an 8-week acclimation period during which they were given a commercial diet (EWOS Dynamic S, Cargill Inc., MN, USA). After the completion of the acclimation period, fish were switched from the commercial feed and fed with the experimental diets (4 tanks diet^−1^) for 12 weeks. At all stages, fish were fed to satiation overnight using automatic feeders (AVF6 Vibrating Feeders, Pentair Aquatic Eco-Systems, Inc., Florida, USA). Uneaten pellets were collected every morning, and the daily amount of feed was adjusted based on the number of uneaten pellets. All tanks were supplied with 12°C flow-through filtered seawater at 12 l min^−1^; the dissolved oxygen level was ~10 mg l^−1^, and the photoperiod was maintained at 24-h light. Mortalities were weighed and recorded throughout the trial. Ethical treatment of fish in this experiment was carried out in accordance with the guidelines of the Canadian Council on Animal Care, and approved by the Institutional Animal Care Committee of Memorial University of Newfoundland (protocol # 16-75-MR).

### Sample Collection

Salmon samples were collected from 10 opportunistically netted fish (each from a different tank) at week 0 (the day before fish were exposed to the experimental diets), and from 5 fish per tank at week 12. Sampling occurred after the fish were starved for 24 h and euthanized with an overdose of MS-222 (400 mg l^−1^; Syndel Laboratories, Vancouver, BC, Canada). For lipid analyses, head kidney samples were collected in 15 ml glass test tubes that had been rinsed three times with methanol, followed by three rinses with chloroform. Samples were stored on ice during sampling, their wet weights recorded, and they were covered with 2 ml of chloroform (HPLC-grade). Finally, the glass test tubes were filled with nitrogen, the Teflon-lined caps were sealed with Teflon tape, and samples were stored at −20°C. For gene expression analyses, head kidney (50–100 mg sample^−1^) tissues were collected in 1.5 ml nuclease-free tubes, flash-frozen in liquid nitrogen, and stored at −80°C until RNA extractions were performed.

### Lipid Extractions

Lipid samples were extracted according to Parrish (1999). Samples were homogenized in a 2:1 mixture of ice-cold chloroform:methanol using a Polytron PCU-2-110 homogenizer (Brinkmann Instruments, Rexdale, ON, Canada). Chloroform-extracted water was added to bring the ratio of chloroform:methanol:water to 8:4:3. Each sample was sonicated for 4 min in an ice bath and centrifuged at 3,000 rpm for 2 min at room temperature. The bottom, organic layer was removed using a double pipetting technique, placing a 2 ml lipid-clean glass Pasteur pipette inside a 1 ml glass pipette, in order to remove the organic layer without disturbing the top, aqueous layer. Chloroform was then added back to the extraction test tube, and the entire procedure was repeated three times. All organic layers were pooled into a separate lipid-clean vial. Finally, samples were concentrated under a flow of nitrogen gas.

### Lipid Class Separation

Iatroscan Mark 6 TLC-FID (thin-layer chromatography-flame ionization detector) (Mitsubishi Kagaku Iatron, Inc., Tokyo, Japan), and a three-step development method were used to determine the lipid class composition (Parrish, 1987). Lipid extracts and standards were applied to the Chromarods and focused to a narrow band using 100% acetone. The first development system was hexane:diethyl ether:formic acid (98.95:1.0:0.05). The rods were developed for 25 min, removed from the system for 5 min to dry, and replaced for 20 min. The second development was for 40 min in hexane:diethyl ether:formic acid (79:20:1). The final development system had two steps–the first was in 100% acetone for two 15 min time periods, followed by two 10 min periods in chloroform:methanol:chloroform extracted water (5:4:1). Before using each solvent system, the rods were dried in a constant humidity chamber. After each development system, the rods were partially scanned in the Iatroscan, and the data were collected using Peak Simple software (SRI Instruments, version 3.67, Torrance, CA, USA). The Chromarods were calibrated using standards from Sigma Chemicals (Sigma Chemicals, St. Louis, MO, USA).

### Fatty Acid Methyl Ester (FAME) Derivatization

Lipid extracts (50 μl) were transferred into lipid-clean 15 ml glass vials and concentrated under a flow of nitrogen to complete dryness. Then, 1.5 ml of methylene chloride and 3 ml of Hilditch reagent (1.5 sulfuric acid: 98.5 anhydrous methanol) were added to each sample vial, and this was followed by a brief vortexing and 4 min sonication (Fisher Scientific FS30, Pittsburgh, PA, USA). Vials were then filled with nitrogen, capped and heated at 100°C for 1 h. Subsequently, 0.5 ml of saturated sodium bicarbonate solution and 1.5 ml of hexane were added to each vial, followed by a brief vortexing, and then the upper, organic layer was removed into a separate lipid-clean glass vial. Each sample was then dried and refilled with ~0.5 ml of hexane. Finally, vials were filled with nitrogen, capped, sealed with Teflon tape, and sonicated for 4 min to resuspend the fatty acids. All solvents were OmniSolv grade (VWR International, Mississauga, Ontario, Canada).

All FAMEs were analysed on a HP 6890 GC-FID (gas chromatograph-flame ionization detector) system equipped with a 7683 autosampler (Agilent Technologies Canada Inc., Mississauga, Ontario, Canada). Additional details regarding the GC conditions (e.g., column length, diameter, temperature, carrier gas) are described in Hixson et al. (2017). Fatty acid peaks were identified against known standards (PUFA 1, PUFA 3, BAME and a Supelco 37 component FAME mixture: Sigma-Aldrich Canada Ltd., Oakville, Ontario, Canada). Finally, chromatograms were integrated using the Varian Galaxie Chromatography Data System, version 1.9.3.2 (Walnut Creek, CA, USA). FA data were expressed as area percentage of FAME.

### RNA Extraction, DNase Treatment, Column Purification and cDNA Synthesis

Head kidney samples were homogenized in TRIzol reagent (Invitrogen, Carlsbad, CA, USA), using the TissueLyser II system at 25 Hz for 2.5 min, using 5 mm stainless steel beads (QIAGEN, Mississauga, ON, Canada), and subjected to RNA extraction. This was followed by DNaseI treatment and column purification using the RNase-free DNase Set (QIAGEN) and the RNeasy Mini Clean-up Kit (QIAGEN), respectively. All procedures were conducted according to manufacturer instructions and as described in Xue et al. (2015). RNA integrity was verified by 1% agarose gel electrophoresis, and RNA purity and quantity were assessed by Nanodrop UV spectrophotometry (Thermo Fisher Scientific, Waltham, MA, USA). DNaseI-treated and column-purified RNA samples had A260/280 and A260/230 ratios of 1.8–2.3 (data not shown). All cDNAs were prepared by reverse transcription of 1 μg of DNaseI-treated, column-purified total RNA for each sample, using 1 μl of random primers (250 ng; Invitrogen), 1 μl of dNTPs (0.5 mM final concentration; Invitrogen), 4 μl of 5X first-strand buffer (1×final concentration; Invitrogen), 2 μl of DTT (10 mM final concentration; Invitrogen), and 1 μl of Moloney murine leukemia virus (M-MLV) reverse transcriptase (RT) (200 U; Invitrogen) at 37°C for 50 min. The total reaction volume was 20 μl, following manufacturer instructions, and as described in Xue et al. (2015). Finally, all cDNAs were diluted 40 times with nuclease-free water (Invitrogen) prior to real-time quantitative polymerase chain reaction (qPCR).

### QPCR Analysis

Expression of 25 genes of interest (GOI) (Table 1) related to FA and eicosanoid metabolism was quantified by qPCR using head kidney cDNA templates from fish fed the five dietary treatments at week 12. For each dietary treatment, eight individuals (two from each tank) were used in the qPCR study. Only head kidney samples from fish that had specific growth rates within 1.5 standard deviations below and above the mean value of each tank were selected for the qPCR study, in order to reduce biological variability. The sequences of all primer pairs used in qPCR analyses, GenBank accession numbers of sequences used for primer design, and other details are presented in Table 1. Notably, primers for the transcripts *fadsd6a, elovl2*, and *elovl5b* (GenBank accession numbers AY458652, FJ237532 and FJ237532, respectively) failed quality testing due to low transcript expression levels, and thus were not included in the qPCR study. Each primer pair was quality-tested to verify that a single product was amplified with no primer dimers, and included standard curves and dissociation curves, as described in Rise et al. (2010) and Booman et al. (2011). In brief, the amplification efficiency (Pfaffl, 2001) of each primer pair was determined using a 5-point 1:3 dilution series starting with cDNA representing 10 ng of input total RNA. Two pools were generated (i.e., 0.3% EPA+DHA↑ω6 and 0.3% EPA+DHA↑ω3), and each pool consisted of 8 fish (with each fish contributing an equal quantity to the pool). The reported primer pair amplification efficiencies were an average of the two pools, except if one pool showed poor efficiency or spacing due to low expression levels (i.e., the 0.3% EPA+DHA↑ω3 pool was used for *fadsd6b* and *fadsd6c*, while the 0.3% EPA+DHA↑ω6 pool was used for *lxrb*).

**Table 1:** qPCR primers.

**Gene name (symbol)**	**Nucleotide sequence (5'−3')^**a**^**	**Amplification efficiency (%)**	**Amplicon size (bp)**	**GenBank Accession number**
Elongation of very long chain fatty acids 4 b (*elovl4b*)^b^	F:TAGCAGAGTTGGGGATCAGC	99.4	101	XM_014144655
	R:CGAGATTTAGGTGCGTGTACTG			
Elongation of very long chain fatty acids 5 a (*elovl5a*)^c^	F:CAGTGTGGTGGGGACAAAG	90.2	115	AY170327
	R:TTCCCTCATGGACAAGCA			
Delta-5 fatty acyl desaturase (*fadsd5*)^c^	F:GTCTGGTTGTCCGTTCGTTT	89.0	135	AF478472
	R:GAGGCGATCAGCTTGAGAAA			
Delta-6 fatty acyl desaturase b (*fadsd6b*)^c^	F:TGACCATGTGGAGAGTGAGG	90.1	95	NM001172281
	R:CCAAAGCCAAGGCCTCTAGT			
Delta-6 fatty acyl desaturase c (*fadsd6c*)	F:CCAGTTGGAATCACGTACTGC	102.7	162	NM001171780
	R:GTGTGTCTCCCAGGACGAAG			
Sterol regulatory element-binding protein 1 (*srebp1*)^c^	F:TCAACAAGTCGGCAATTCTG	103.1	100	HM561860
	R:GACATCTTCAGGGCCATGTT			
Sterol regulatory element-binding protein 2 (*srebp2*)^c^	F:GAGTGCTGAGGAAAGCCATC	103.1	129	HM561861
	R:TCTCCACATCGTCAGACAGC			
Liver X receptor a (*lxra*)^c^	F:GGGCAAGATGGACAGATCAT	102.6	126	FJ470290
	R:CCTCACCAGGACCAACATCT			
Liver X receptor b (*lxrb*)^d^	F:CTCGCCTGTGTTCCTGTTTT	92.6	103	GE770391
	R:GAAACGCAAGACCTTCTGCT			
Peroxisome proliferator-activated receptor alpha a (*pparaa*)^b^	F:CCCTGGTGGCTAAGATGGT	103.1	132	XM_014124067
	R:AGACTTGGCGAACTCGGTTA			
Peroxisome proliferator-activated receptor beta 1 (*pparb1*)	F:CAGCTGATCAACGGTACGAC	93.4	112	NM001123635
	R:TGCTCTTGGCAAACTCAGTG			
Peroxisome proliferator-activated receptor beta 2 a (*pparb2a*)^c^	F:CCGTTTGTGATCCATGATGT	103.7	128	NM001123559
	R:GTGCACTGACAGCGGTAAAA			
Fatty acid synthase a (*fasa*)^c^	F:GGAGGGCACAATGGAGTAAA	97.7	136	DW563978
	R:TGAGACAGTGAATCGGATGG			
Fatty acid synthase b (*fasb*)^c^	F:TGCCATACAAGTGATGTCCTG	99.1	105	EG872804
	R:AGTGGGCACCAAACATGAAC			
Acyl-coenzyme A oxidase 1 (*acox1*)^b^	F:GTGCACCTACGAGGGAGAGA	99.9	111	DW555884
	R:TAGGACACGATGCCACTCAG			
Carnitine palmitoyltransferase 1 a (*cpt1a*)^b^	F:CGGTGGCAGATGATGGATATG	104.6	82	XM_014176428
	R:GAGTGCTTGCTGGAGATGTG			
Cytosolic phospholipase A2 (*cpla2*)	F:GACGTGGCAGATTCAGACAA	92.3	147	NM_001141333
	R:GAACCAGAGAGATGGCAGGT			
Cyclooxygenase-1 (*cox1*)^c^	F:CTCATGAGGGTGGTCCTCAC	104.1	135	BT045745
	R:AGGCACAGGGGGTAGGATAC			
Cyclooxygenase-2 (*cox2*)^e^	F:ACCTTTGTGCGAAACGCTAT	97.0	113	AY848944
	R:GAGTAGGCCTCCCAGCTCTT			
Arachidonate 5-lipoxygenase a *(5loxa*)^c^	F:CTGCTCACCATGCTGCTGTC	97.0	93	NM001139832
	R:GTGTGGGAGGAGGCTTCC			
Arachidonate 5-lipoxygenase b (*5loxb*)^c^	F:ACTGCTGTGGGTTTCCCAAG	102.6	98	DW555519
	R:GACAGCAGCGTGATGTGCAG			
Prostaglandin-D synthase (*pgds*, alias *lipocalin-type pgds*)^c^	F:GGTGCTCAACAAGCTCTACA	90.3	114	BT048787
	R:GCAGGAAAGCGATGTTGTCA			
Prostaglandin E synthase 2-like (*ptges2*)	F:TTCTGCGCTGTTACCCAGAG	99.8	112	XM_014171682/014160437
	R:GTACATCGTCTGACCTTCAG			
Prostaglandin E synthase 3 (*ptges3*)	F:TGGCCTAGGCTAACGAAAGA	97.7	101	BT056895
	R:TTGCCTAGTTCCTCGTCTGAG			
Leukotriene A4 hydrolase (*lkha4*, alias *lta4h*)	F:AAGGTCTCCAAGGTAACAGC	94.8	97	NM_001140120
	R:AATGGCAGTGTGATCTCCAA			
**Eukaryotic translation initiation factor 3 subunit D (*****eif3d*****)**^**e**^	F:CTCCTCCTCCTCGTCCTCTT	94.2	105	GE777139
	R:GACCCCAACAAGCAAGTGAT			
**Polyadenylate-binding protein cytoplasmic 1 (*****pabpc1*****)**^**e**^	F:TGACCGTCTCGGGTTTTTAG	93.6	108	EG908498
	R:CCAAGGTGGATGAAGCTGTT			

a *F is forward and R is reverse primer*.

b *Primers that were previously published in Emam et al. (2020)*.

c *Primers that were previously published in Katan et al. (2019)*.

d *Primers that were previously published in Hixson et al. (2017)*.

e *Primers that were previously published in Caballero-Solares et al. (2017). Normalizer genes are in bold font*.

To select the most suitable normalizer genes, six candidate normalizers were tested based on our previous qPCR studies (*rpl32, eef1*α*-1, eef1*α*-2, actb, eif3d, pabpc1*) (Xue et al., 2015; Caballero-Solares et al., 2017), and salmon literature on reference genes (*actb, eef1*α*-1, eef1*α*-2*) (Olsvik et al., 2005). Their qPCR primers were quality-tested as mentioned above. Half of the fish population involved in the qPCR study was utilized for normalizer testing (i.e., 20 total which consisted of 4 fish per treatment). Cycle threshold (C_T_) values were measured using cDNA corresponding to 5 ng of input total RNA. Expression stability was then analysed using the geNorm algorithm (Vandesompele et al., 2002). *Eif3d* and *pabpc1* were shown to be the most stable (i.e., geNorm M-values of 0.23 and 0.22, respectively) among the six candidate genes, and therefore were selected as normalizers.

All qPCR amplifications were performed in a total reaction volume of 13 μl and consisted of 4 μl of cDNA (5 ng input total RNA), 50 nM each of forward and reverse primer, 1×Power SYBR Green PCR Master Mix (Applied Biosystems), and nuclease-free water (Invitrogen). QPCR reactions, including no-template controls, were performed in technical triplicates using the ViiA 7 Real-Time PCR System (384-well format) (Applied Biosystems, Foster City, CA, USA) and the Power SYBR Green I dye chemistry. The Real-Time analysis program consisted of 1 cycle of 50°C for 2 min, 1 cycle of 95°C for 10 min, followed by 40 cycles (of 95°C for 15 s and 60°C for 1 min), with the fluorescence signal data collection after each 60°C step. When a C_T_ value within a triplicate was >0.5 cycle from the other two values, it was considered to be an outlier, discarded and the average C_T_ of the remaining two values was calculated. The relative quantity (RQ) of each GOI was calculated using a qBase relative quantification framework (Hellemans et al., 2007; Booman et al., 2014) with primer amplification efficiencies incorporated (Table 1). The expression levels of each GOI were normalized to both normalizer genes, and the sample with the lowest normalized expression was used as the calibrator sample (i.e., RQ = 1.0) for each GOI (Rise et al., 2015). Transcript expression data are presented as RQ values relative to the calibrator. QPCR fold-change values were calculated by dividing the mean RQ value of each dietary treatment by that of the 0.3% EPA+DHA↑ω6 fish.

### Statistical Analyses

#### Tissue Lipid Composition and qPCR Data

Nested general linear models were used with tank nested in diet (Minitab 17 Statistical Software, State College, PA, USA). This was followed by Tukey *post-hoc* tests (*p* < 0.05) to identify significant differences among treatments at week 12 (Minitab 17 Statistical Software). However, when a significant tank effect was identified (*p* < 0.05), a one-way ANOVA followed by Tukey *post-hoc* tests was performed (Minitab 17 Statistical Software, State College, PA, USA). To identify significant differences in head kidney lipid composition between week 0 (i.e., initial) and week 12, a one-way ANOVA followed by Tukey *post-hoc* tests was used. To show the effects of dietary EPA+DHA (i.e., 0.3 and 1%) and ω6:ω3 (i.e., high ω6 and high ω3) factors on the transcript expression of each GOI, a two-way ANOVA was performed (Minitab 17 Statistical Software). The 1.4% EPA+DHA/balanced treatment was not included in this analysis since it was not formulated following the 2 × 2 factorial design of the other four treatments (i.e., 0.3 or 1.0% EPA+DHA *vs*. high ω6 or high ω3).

For the qPCR analysis, each dietary treatment was tested for outliers using Grubb's test (*p* < 0.05). In total, 7 RQ values were identified as statistical outliers in the entire dataset (i.e., out of 1,000 values comprising all samples and all GOIs), and excluded from the study. All GOIs had a sample size of 7–8 per dietary treatment. Finally, residuals were tested to verify normality, independence and homogeneity of variance. Normality was examined using the Anderson-Darling test. If the test failed (*p* < 0.05), a one-way ANOVA on ranks was performed, and followed by a Kruskal-Wallis test (SigmaPlot, Systat Software, Inc., Version 13, San Jose, CA, USA). In all cases, differences were considered statistically significant when *p* < 0.05.

#### Multivariate and Pearson Correlation Analyses

Principal coordinate analysis (PCoA) was used to describe head kidney lipid and FA composition in the five dietary treatments (PRIMER, Plymouth Routines in Multivariate Ecological Research; PRIMER-E Ltd., version 6.1.15, Ivybridge, UK). A similarity matrix was used, and the first two PCO axes (i.e., PCO1, PCO2) were plotted. SIMPER (Similarity of Percentages Analysis) was performed to quantify differences among treatments in lipid and FA data, while PERMANOVA (Permutational Multivariate Analysis of Variance) was used to perform pairwise tests between treatments (9,999 permutations). In all cases, the non-parametric Bray-Curtis similarity was used.

Pearson correlation analysis was performed to identify relationships between transcript expression (i.e., qPCR RQ data) and tissue lipid composition (i.e., % FA and lipid classes) in the head kidney, using individual fish. All GOIs and lipid classes [i.e., triacylglycerols (TAG), sterols (ST) and phospholipids (PL)] were used in the correlation analysis. However, transcripts and lipid classes with no significant correlations were removed. Individual saturated fatty acids (SFA) and monounsaturated fatty acids (MUFA) that accounted for >5% of the total FA, and all ω6 and ω3 FA were included in the analysis. IBM SPSS (IBM SPSS Statistics, Version 25, Armonk, New York, USA) was used for the correlation analyses. In order to separately group transcripts and lipid composition (% fatty acids and lipid classes), hierarchical clustering was used with group average in PRIMER (Version 6.1.15, Ivybridge, UK).

## Results

### Head Kidney Lipid Composition

The predominant lipid class in the head kidney was TAG (46.6–52.0%), followed by PL (22.8–28.6%), and ST (18.4–23.7%) (Table 2). No significant differences were observed among dietary treatments in any lipid class or in total lipids (mg g^−1^) at week 12 (*p* = 0.09–0.72). ST showed a decrease from week 0 (initial) to week 12 in all treatments (*p* < 0.0001, Table 2).

**Table 2:** Lipid and fatty acid composition (%) of Atlantic salmon head kidney^a^ before (Initial) and after 12 weeks of feeding diets with different ratios of ω6:ω3 and levels of EPA+DHA.

	**Initial**	**0.3% EPA+DHA**	**0.3% EPA+DHA**	**1% EPA+DHA**	**1% EPA+DHA**	**1.4% EPA+DHA**
		**↑ω6**	**↑ω3**	**↑ω6**	**↑ω3**	**Balanced**
**Lipid classes composition (% of total lipid)**						
TAG (%)^b^	38.9 ± 18.7	46.6 ± 12.5	48.5 ± 12.8	47.8 ± 9.8	47.0 ± 15.9	52.0 ± 14.7
ST (%)^c^	36.7 ± 15.2	23.7 ± 6.9	20.5 ± 7.2	23.5 ± 6.0	21.8 ± 6.8	18.4 ± 6.8
PL (%)^d^	23.5 ± 11.4	27.2 ± 14.1	28.6 ± 11.2	22.8 ± 9.1	28.2 ± 12.3	25.4 ± 11.0
TL (mg g^−1^)^e^	32.1 ± 12.0	26.9 ± 7.2	33.8 ± 9.0	31.9 ± 8.3	35.1 ± 14.7	36.2 ± 13.4
**Fatty acid composition (% of total FA)**						
14:0	2.4 ± 0.5	0.9 ± 0.4^*a*^	1.1 ± 0.3^*a*^	1.4 ± 0.3^*a*^^*b*^	1.7 ± 0.8^*b*^^*c*^	2.1 ± 0.9^c^
16:0	15.4 ± 2.0	14.9 ± 1.2	14.1 ± 0.8	15.6 ± 3.2	14.9 ± 3.7	14.8 ± 2.7
16:1ω7	5.6 ± 1.0	2.1 ± 0.8^*a*^	2.9 ± 0.5^*b*^^*c*^	2.4 ± 0.8^*a*^^*b*^	3.1 ± 1.0^*b*^^*c*^	3.4 ± 0.9^*c*^
18:0	5.0 ± 0.7	5.6 ± 1.0^a^	5.2 ± 0.4^a^^b^	5.7 ± 1.9^a^	4.9 ± 0.5^a^^b^	4.4 ± 0.3^b^
18:1ω9	31.5 ± 4.7	20.7 ± 3.1^*a*^	24.8 ± 2.3^*b*^^*c*^	20.0 ± 2.9^*a*^	22.6 ± 2.5^*a*^^*b*^	25.7 ± 2.5^*c*^
18:1ω7	3.4 ± 0.4	2.2 ± 0.2^*a*^	2.3 ± 0.1^*a*^^*b*^	2.4 ± 0.3^*a*^^*b*^	2.4 ± 0.2^*b*^	3.0 ± 0.1^*c*^
18:2ω6 (LNA)	10.1 ± 1.1	19.3 ± 3.1^*a*^	11.6 ± 1.2^c^	16.3 ± 3.6^*b*^	10.5 ± 1.4^c^	9.7 ± 1.0^c^
18:3ω6	0.3 ± 0.1	0.9 ± 0.2^*a*^	0.5 ± 0.1^*b*^	0.5 ± 0.1^*b*^	0.3 ± 0.1^c^	0.3 ± 0.1^c^
18:3ω3 (ALA)	1.1 ± 0.3	1.9 ± 0.4^a^	6.5 ± 1.2^*b*^	1.7 ± 0.5^a^	6.3 ± 1.5^*b*^	1.7 ± 0.3^a^
18:4ω3	0.6 ± 0.2	0.7 ± 0.2^a^	1.8 ± 0.4^*c*^	0.7 ± 0.2^a^	1.5 ± 0.3^*b*^	0.9 ± 0.2^*a*^
20:1ω11	0.5 ± 0.1	0.2 ± 0.1^*a*^	0.2 ± 0.3^a^	1.0 ± 0.3^*b*^	0.9 ± 0.3^*b*^	1.5 ± 0.4^*c*^
20:1ω9	2.9 ± 0.5	1.2 ± 0.4^*a*^	1.4 ± 0.2^*a*^^*b*^	1.6 ± 0.3^*b*^	1.6 ± 0.5^*b*^	2.1 ± 0.4^*c*^
20:2ω6	0.6 ± 0.1	1.4 ± 0.3^*a*^	0.7 ± 0.1^b^	1.2 ± 0.4^*a*^	0.6 ± 0.2^b^	0.8 ± 0.1^b^
20:3ω6 (DGLA)	0.4 ± 0.1	2.6 ± 0.7^*a*^	1.0 ± 0.2^*c*^	1.5 ± 0.5^*b*^	0.7 ± 0.2^d^	0.7 ± 0.1^c^^d^
20:4ω6 (ARA)	1.2 ± 0.8	4.3 ± 1.7^*a*^	2.5 ± 0.6^*b*^	2.4 ± 0.8^*b*^	2.0 ± 0.7^b^	1.9 ± 0.6^b^
20:3ω3	0.1 ± 0.04	0.1 ± 0.03^a^	0.4 ± 0.1^*b*^	0.2 ± 0.03^a^	0.4 ± 0.1^*b*^	0.2 ± 0.02^*a*^
20:4ω3	0.4 ± 0.2	0.4 ± 0.1^a^	1.0 ± 0.2^*c*^	0.6 ± 0.1^a^^b^	0.9 ± 0.3^*c*^	0.7 ± 0.2^*b*^
20:5ω3 (EPA)	2.9 ± 1.7	2.6 ± 0.6^a^	3.7 ± 0.7^b^	3.8 ± 1.2^b^	4.4 ± 1.3^*b*^	4.3 ± 1.1^*b*^
22:1ω11	2.4 ± 0.4	0.7 ± 0.3^*a*^	0.8 ± 0.3^*a*^	1.4 ± 0.3^*b*^	1.4 ± 0.5^*b*^	1.8 ± 0.5^*c*^
22:4ω6	0.1 ± 0.05	0.2 ± 0.1^*a*^	0.1 ± 0.01^b^	0.2 ± 0.1^a^^b^	0.1 ± 0.1^b^	0.2 ± 0.2^a^^b^
22:5ω6 (ω6 DPA)	0.1 ± 0.1	0.4 ± 0.1^*a*^	0.2 ± 0.05^b^	0.2 ± 0.04^b^	0.2 ± 0.05^b^	0.2 ± 0.04^b^
22:5ω3 (ω3 DPA)	1.0 ± 0.5	0.8 ± 0.3^a^	1.0 ± 0.1^a^^b^^c^	1.0 ± 0.4^a^^b^	1.1 ± 0.4^b^^c^	1.3 ± 0.4^c^
22:6ω3 (DHA)	6.8 ± 4.3	12.4 ± 2.9	12.8 ± 3.0	13.5 ± 3.4	13.6 ± 3.8	14.0 ± 3.8
ΣSFA^f^	23.8 ± 2.9	22.3 ± 2.2	21.2 ± 1.0	23.8 ± 5.6	22.4 ± 4.9	22.2 ± 3.7
ΣMUFA^g^	48.5 ± 6.9	28.3 ± 4.7^*a*^	33.8 ± 3.0^*a*^^*b*^	30.7 ± 4.5^*a*^^*b*^	33.7 ± 3.6^*b*^	39.3 ± 3.7^*c*^
ΣPUFA^h^	27.3 ± 8.8	49.0 ± 4.5^*a*^	44.6 ± 2.4^*a*^^*b*^	45.1 ± 8.3^*a*^^*b*^	43.6 ± 5.0^*b*^	38.1 ± 4.3^*c*^
Σω3	13.1 ± 7.2	19.1 ± 3.0^*a*^	27.4 ± 2.9^*c*^	21.7 ± 5.0^*a*^^*b*^	28.5 ± 4.8^*c*^	23.4 ± 4.4^*b*^
Σω6	12.9 ± 1.9	28.9 ± 3.4^*a*^	16.4 ± 0.8^*c*^	22.1 ± 4.6^*b*^	14.3 ± 1.2^c^^d^	13.6 ± 0.5^d^
ω6:ω3	1.2 ± 0.7	1.5 ± 0.3^*a*^	0.6 ± 0.1^*c*^	1.0 ± 0.2^b^	0.5 ± 0.1^*c*^	0.6 ± 0.1^*c*^
EPA+DHA	9.7 ± 6.0	15.0 ± 3.3	16.5 ± 3.4	17.3 ± 4.4	18.0 ± 4.8	18.3 ± 4.6
DHA:EPA	2.4 ± 0.2	4.9 ± 1.1^*a*^	3.4 ± 0.6^*b*^	3.7 ± 0.7^*b*^	3.2 ± 0.7^b^	3.3 ± 0.5^*b*^
EPA:ARA	2.4 ± 0.6	0.7 ± 0.3^*a*^	1.6 ± 0.3^*b*^	1.6 ± 0.3^*b*^	2.2 ± 0.4^c^	2.3 ± 0.5^c^

a *Mean (n = 10–20) ± standard deviation (SD). Different superscripts in the same row indicate significant differences among treatments at week 12. Underlining indicates values that are significantly different to week 0 (Initial) (p < 0.05)*.

b *Triacylglycerol*.

c *Sterols*.

d *Phospholipids*.

e *Total lipids*.

f *Total saturated fatty acids*.

g *Total monounsaturated fatty acids*.

h *Total polyunsaturated fatty acids*.

Individual SFA showed differences among treatments (i.e., 14:0 and 18:0; *p* < 0.0001 and 0.001, respectively) at week 12. The 1.4% EPA+DHA/balanced treatment had higher 14:0 than the 0.3% EPA+DHA↑ω6, 0.3% EPA+DHA↑ω3 and 1.0% EPA+DHA↑ω6 fed fish. Lower 18:0 levels were observed in the 1.4% EPA+DHA/balanced compared to the high ω6 (i.e., with 0.3% and 1.0% EPA+DHA) treatments. However, 16:0 and total SFA (ΣSFA) were similar among dietary treatments (*p* = 0.60 and 0.45, respectively) at week 12 (Table 2).

Salmon fed the 1.4% EPA+DHA/balanced diet had the highest level of total MUFA (ΣMUFA), while the 1% EPA+DHA↑ω3 fed fish showed higher ΣMUFA than the 0.3% EPA+DHA↑ω6 fish at week 12 (*p* < 0.0001). These differences were also observed in some individual MUFA (i.e., 18:1ω7, 20:1ω11, 20:1ω9, 22:1ω11; *p* < 0.0001). The 0.3% EPA+DHA↑ω3 fish had higher 18:1ω9 compared to fish fed the high ω6 diets (i.e., 0.3 and 1% EPA+DHA), and this FA was higher in the 1.4% EPA+DHA/balanced than the other treatments (not including 0.3% EPA+DHA↑ω3; *p* < 0.0001). All treatments showed a significant decrease in ΣMUFA, 18:1ω9 and other MUFA (i.e., 16:1ω7, 18:1ω7, 20:1ω9, 22:1ω11) when comparing week 12 to week 0 (*p* < 0.0001).

The levels of LC-PUFA precursors 18:2ω6 and 18:3ω3 reflected experimental diet levels (i.e., highest 18:2ω6 in the high ω6 fed salmon; highest 18:3ω3 in the high ω3 fed salmon; *p* < 0.0001; Table 2). Further, compared with week 0, 18:2ω6 and 18:3ω3 increased after 12 weeks of feeding high ω6 (i.e., 0.3% EPA+DHA↑ω6 and 1% EPA+DHA↑ω6) and high ω3 (0.3% EPA+DHA↑ω3 and 1% EPA+DHA↑ω3) diets, respectively (*p* < 0.0001; Table 2). The intermediate ω6 (18:3ω6, 20:2ω6 and 20:3ω6) and ω3 PUFA (18:4ω3, 20:3ω3 and 20:4ω3), and the ω6:ω3 ratios were different among treatments at week 12 (*p* < 0.0001) and responded to dietary changes in ω6 and ω3 PUFA. The proportion of 20:4ω6 was highest in fish fed 0.3% EPA+DHA↑ω6 diet (*p* < 0.0001), while no significant differences were observed among the remaining four treatments at week 12. In contrast, the 0.3% EPA+DHA↑ω6 fish showed the lowest 20:5ω3 (*p* < 0.0001), and similar levels of this ω3 LC-PUFA were observed among the remaining four treatments (*p* = 0.18) at week 12. Further, after 12 weeks of feeding, no differences were shown among dietary treatments in 22:6ω3 (*p* = 0.53). Compared with week 0, 20:4ω6 showed an increase after 12 weeks of feeding high ω6 (i.e., 0.3 and 1% EPA+DHA) and the 0.3% EPA+DHA↑ω3 diet, while 20:5ω3 increased in the 1% EPA+DHA↑ω3 and 1.4% EPA+DHA/balanced fed fish. All 5 treatments increased in 22:6ω3 at week 12 compared to week 0 (*p* < 0.0001). Fish fed the 0.3% EPA+DHA↑ω6 diet had the highest ω6:ω3, and the high ω6 fish (i.e., 0.3 and 1% EPA+DHA) had higher ω6:ω3 compared to high ω3 (i.e., 0.3 and 1% EPA+DHA) and 1.4% EPA+DHA/balanced fed fish at week 12 (*p* < 0.0001). The DHA:EPA ratio was higher in the 0.3% EPA+DHA↑ω6 fed fish compared with the other four treatments at week 12 (*p* < 0.0001; Table 2). Finally, EPA:ARA was highest in the 1% EPA+DHA↑ω3 and the 1.4% EPA+DHA/balanced treatments, and lowest in the 0.3% EPA+DHA↑ω6 fed fish (*p* < 0.0001).

**Figure 1:**
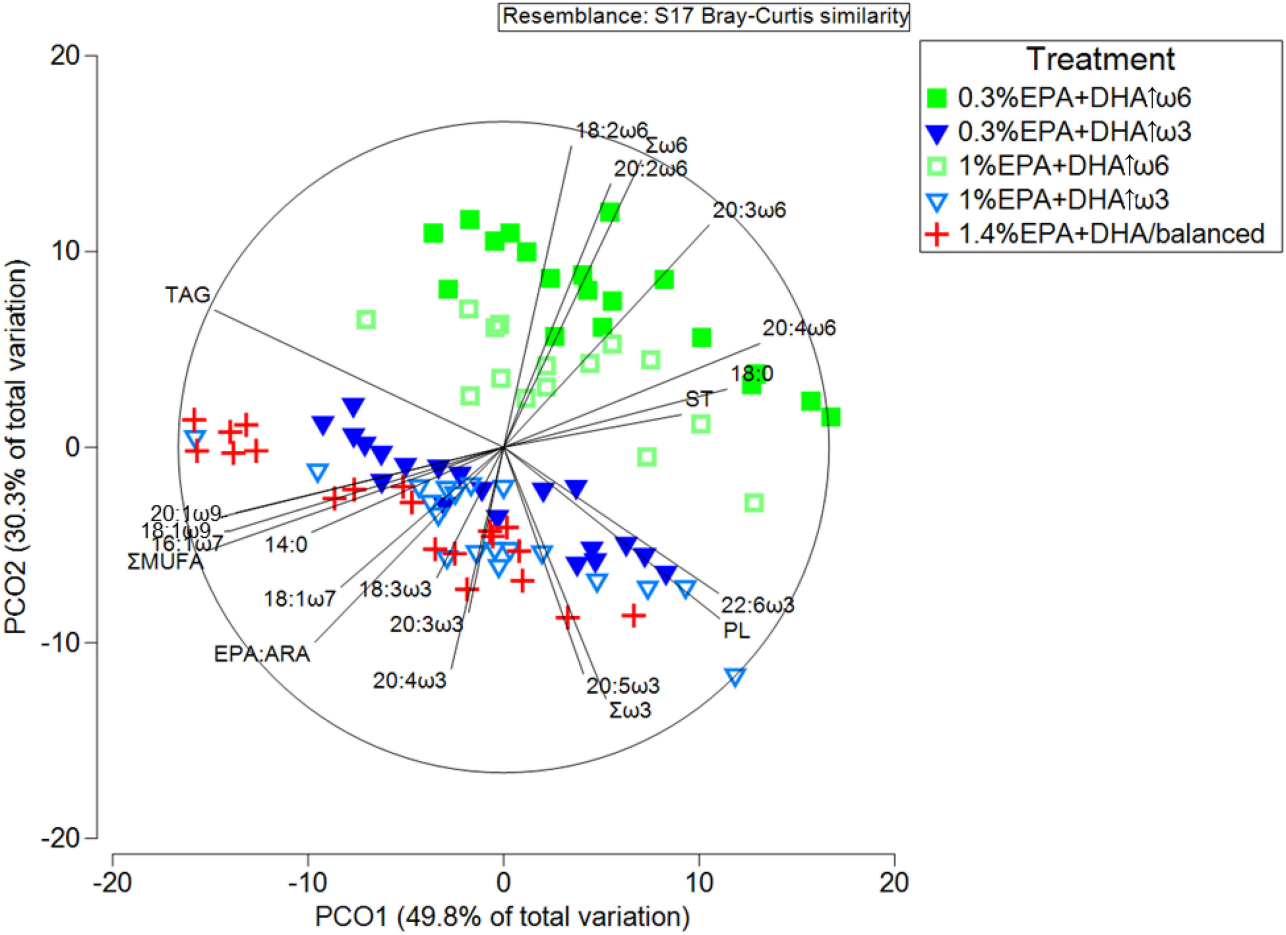
Principal coordinates analysis (PCoA) of lipid and FA composition (%) (*r*^2^ > 0.45) in the head kidney of Atlantic salmon after 12 weeks of feeding diets with different ratios of ω6:ω3 and levels of EPA+DHA. PL, ST, and TAG represent the lipid classes phospholipid, sterol and triacylglycerol, respectively. ΣMUFA and EPA:ARA represent total monounsaturated fatty acids and the ratio of eicosapentaenoic (20:5ω3) to arachidonic (20:4ω6) acid, respectively.

PCoA showed a clear separation between the high ω6 and high ω3 treatments (i.e., 0.3% EPA+DHA↑ω6 and 1% EPA+DHA↑ω6 on top, and 0.3% EPA+DHA↑ω3 and 1% EPA+DHA↑ω3 on the bottom; Figure 1). The 1.4% EPA+DHA/balanced fed fish clustered with the high ω3 treatments. PCO1 and PCO2 accounted for 49.8 and 30.3% of the variability, respectively. PERMANOVA pairwise tests showed that the 0.3% EPA+DHA↑ω3 were not different from 1% EPA+DHA↑ω3 fed fish [p(perm) = 0.12]. However, all other treatments were significantly different from each other [p(perm) = 0.0001–0.003] (Supplementary Table 3). PCoA vectors showed that fish fed high ω6 diets (particularly 0.3% EPA+DHA↑ω6) were associated with 18:2ω6, 20:2ω6, 20:3ω6, 20:4ω6, and Σω6, while the high ω3 and 1.4% EPA+DHA/balanced diet fed fish were associated with 18:3ω3, 20:3ω3, 20:4ω3, 20:5ω3, Σω3, EPA:ARA, the MUFA 18:1ω7, 16:1ω7, 18:1ω9 and 20:1ω9, as well as ΣMUFA (Figure 1). The lipid classes ST and PL clustered with the high ω6 and high ω3 (including 1.4% EPA+DHA/balanced) treatments, respectively.

SIMPER analysis indicated that the dissimilarity between 0.3% EPA+DHA↑ω6 and 0.3% EPA+DHA↑ω3, and between 0.3% EPA+DHA↑ω6 and 1% EPA+DHA↑ω6 were mainly caused by Σω6, Σω3, and the lipid classes TAG, PL and ST (Supplementary Table 4). The main FA contributors to the dissimilarities between the remaining high ω6 and high ω3 comparisons were Σω6 and 18:2ω6. Finally, the FA dissimilarity between 1.4% EPA+DHA/balanced and the high ω6 treatments was mainly driven by Σω6, ΣPUFA and ΣMUFA, while ΣPUFA contributed to the FA dissimilarity between 1.4% EPA+DHA/balanced and the high ω3 treatments (Supplementary Table 4). This analysis also showed that the highest dissimilarity in lipid composition profile was between 0.3% EPA+DHA↑ω6 and 1.4% EPA+DHA/balanced fed fish (average dissimilarity = 18.7%), while the lowest dissimilarity was between the two high ω3 (i.e., 0.3% EPA+DHA↑ω3 and 1% EPA+DHA↑ω3; average dissimilarity = 10.0%) and the two high ω6 (i.e., 0.3% EPA+DHA↑ω6 and 1% EPA+DHA↑ω6; average dissimilarity = 11.0%) treatments.

### QPCR Analysis of Transcripts Involved in Fatty Acid Metabolism and Eicosanoid Synthesis

#### Dietary Impacts on Transcript Expression

**Figure 2:**
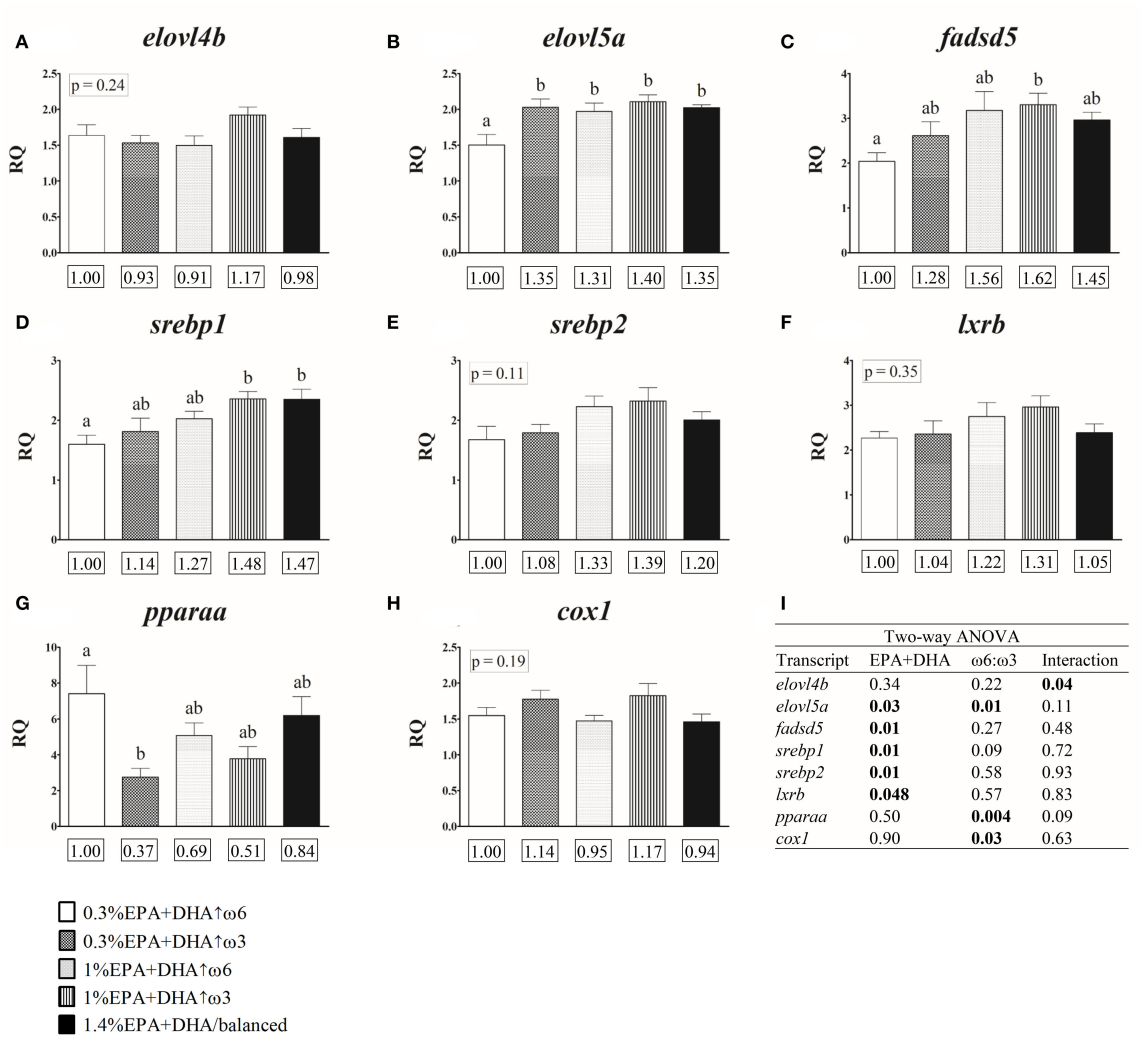
Head kidney qPCR transcripts related to fatty acid metabolism **(A–G)** and eicosanoid synthesis **(H)** in salmon fed diets with different ratios of ω6:ω3 and levels of EPA+DHA for 12 weeks. Transcripts identified as statistically significant in the two-way ANOVA analysis are presented **(A–I)**. Transcript expression data of these and other genes of interest (GOIs) are presented in Supplementary Table 5. Transcript expression values presented as mean relative quantity (RQ) ± SE (*n* = 7–8). Different letters above error bars indicate significant differences among treatments. When differences were not statistically significant (one-way ANOVA and Tukey *post-hoc* tests; *p* > 0.05), *p*-values are shown above error bars. Values below each GOI represent fold-changes relative to the 0.3% EPA+DHA↑ω6 treatment (see Materials and Methods). **(I)** Two-way ANOVA results. Significant differences (*p* < 0.05) are shown in bold font. Two-way ANOVA analysis of the entire set of GOIs is presented in Supplementary Table 6.

One-way ANOVA indicated that salmon fed 0.3% EPA+DHA↑ω3, 1.4% EPA+DHA/balanced, and the 1% EPA+DHA diets had higher expression levels (1.31–1.40-fold; *p* = 0.01) of the transcript *elovl5a* compared with the 0.3% EPA+DHA↑ω6 fed fish (Figure 2B and Supplementary Table 5). The head kidney transcript expression of *fadsd5* was higher in the 1% EPA+DHA↑ω3 compared with the 0.3% EPA+DHA↑ω6 fed fish (1.62-fold; *p* = 0.04; Figure 2C and Supplementary Table 5), while *srebp1* mRNA levels were higher in the 1% EPA+DHA↑ω3 and 1.4% EPA+DHA/balanced treatments compared with the 0.3% EPA+DHA↑ω6 fed fish (1.48- and 1.47-fold, respectively; *p* = 0.02; Figure 2D and Supplementary Table 5). The transcript *pparaa* showed lower expression in the 0.3% EPA+DHA↑ω3 compared with the 0.3% EPA+DHA↑ω6 fed fish (0.37-fold; *p* = 0.02; Figure 2G and Supplementary Table 5). The prostaglandin synthesis-related transcript *ptges3* showed an increasing trend (although not significant) in the 0.3% EPA+DHA↑ω3 compared with the 0.3% EPA+DHA↑ω6 fed fish (1.30-fold; *p* = 0.06; Supplementary Table 5). Finally, the transcript expression of *elovl4b, srebp2* and *lxrb* (Figures 2A,E,F, respectively, and Supplementary Table 5) was not significantly impacted by diet.

#### Effects of EPA+DHA and ω6:ω3 Factors on Transcript Expression

Two-way ANOVA analysis showed a significant interaction between the dietary factor EPA+DHA and ω6:ω3, impacting the transcript expression of *elovl4b* (Figure 2I and Supplementary Table 6). The transcript expression of *elovl5a* was significantly affected by both EPA+DHA and ω6:ω3, while *fadsd5, srebp1, srebp2* and *lxrb* were significantly affected by dietary EPA+DHA levels. Finally, the transcript expression of *pparaa* and *cox1* was significantly affected by the factor ω6:ω3 (Figure 2I and Supplementary Table 6).

### Hierarchical Clustering and Pearson Correlation Analyses

#### Hierarchical Clustering of Head Kidney Transcript Expression and Lipid Composition

**Figure 3:**
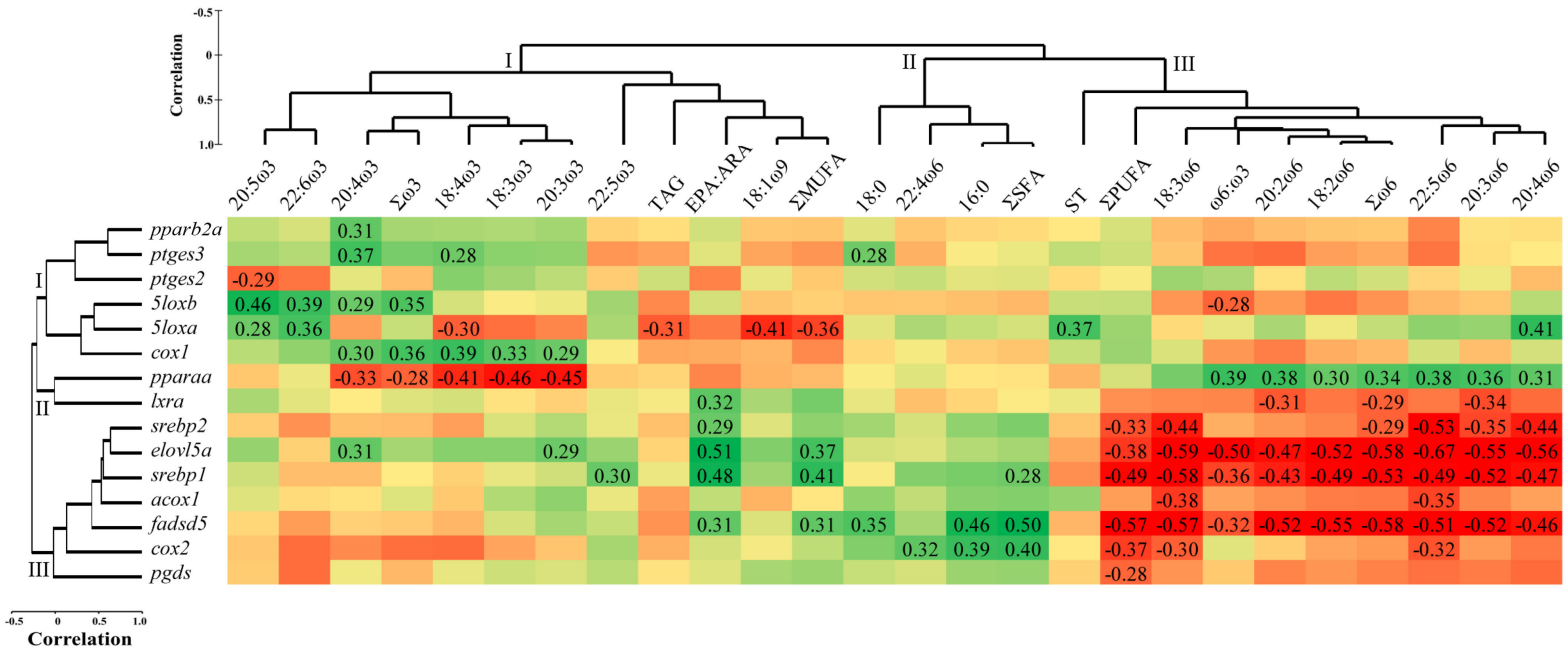
Hierarchical clustering and Pearson correlation matrix of head kidney transcript expression [qPCR relative quantity (RQ) values] and lipid composition (% fatty acids and lipid classes) in Atlantic salmon fed diets with different ratios of ω6:ω3 and levels of EPA+DHA for 12 weeks. Transcripts, fatty acids, and lipid classes that were not significantly correlated were excluded (see Materials and Methods). Statistically significant (*p* < 0.05) correlation coefficients are shown. Red cells signify negative relationships, and green cells signify positive relationships. ΣSFA, ΣMUFA, and ΣPUFA represent total saturated, monounsaturated, and polyunsaturated fatty acids, respectively. 20:5ω3, 22:5ω3, 22:6ω3, and 20:4ω6 represent EPA, DPA, DHA, and ARA, respectively. TAG and ST represent triacylglycerol and sterols, respectively.

Hierarchical clustering of the qPCR analysed transcripts identified three separate clusters (Figure 3). Cluster I consisted of the transcript *pparb2a*, and the eicosanoid metabolism-related transcripts *ptges3, ptges2, 5loxa, 5loxb*, and *cox1*. Cluster II included *pparaa* and *lxra*, while cluster III included the lipid metabolism-related transcripts *srebp2, elovl5a, srebp1, acox1, fadsd5*, and the eicosanoid synthesis-related *cox2* and *pgds*. Cluster analysis of head kidney lipid composition also showed three clusters. Cluster I was composed of the ω3 FA (i.e., 20:5ω3, 22:6ω3, 20:4ω3, 18:4ω3, 18:3ω3, 20:3ω3, and 22:5ω3), Σω3, 18:1ω9, ΣMUFA, EPA:ARA, and the lipid class TAG. Cluster II included 18:0, 16:0, ΣSFA and 22:4ω6. Cluster III showed the ω6 FA (i.e., 18:3ω6, 20:2ω6, 18:2ω6, 22:5ω6, 20:3ω6, 20:4ω6), Σω6, ω6:ω3, as well as ΣPUFA and the lipid class ST (Figure 3).

#### Correlations Between Lipid Composition and Fatty Acid Metabolism Transcripts

The head kidney transcript expression of *pparb2a* was correlated positively with 20:4ω3 (*p* = 0.031; Figure 3), while *pparaa* showed negative correlations with several ω3 FA (i.e., 18:3ω3, 18:4ω3, 20:3ω3, 20:4ω3) and Σω3 (*p* = 0.002–0.044), and positive correlations with several ω6 FA (i.e., 18:2ω6, 20:2ω6, 20:3ω6, 20:4ω6, 22:5ω6), Σω6, and the ratio of ω6:ω3 (*p* = 0.009–0.038). The five transcripts *lxra, srebp2, elovl5a, srebp1*, and *fadsd5* showed positive correlations with EPA:ARA (*p* = 0.001–0.032; Figure 3). The mRNA levels of *elovl5a* also showed positive correlations with 20:3ω3, 20:4ω3 and ΣMUFA (*p* = 0.013–0.044), while *srebp1* correlated positively with 22:5ω3 and ΣMUFA and ΣSFA (*p* = 0.006–0.047). The transcript *fadsd5* correlated positively with ΣMUFA, ΣSFA, and the FA 16:0 and 18:0 (*p* = 0.001–0.032). The same five transcripts (i.e., *lxra, srebp2, elovl5a, srebp1*, and *fadsd5*) and *acox1* correlated negatively with ω6 FA; *lxra* with 20:2ω6, 20:3ω6 and Σω6 (*p* = 0.021–0.042), and *srebp2* with 18:3ω6, 20:3ω6, 20:4ω6, 22:5ω6, as well as Σω6 and ΣPUFA (*p* = 0.0001–0.041). *Elovl5a, srebp1*, and *fadsd5* correlated negatively with 18:2ω6, 18:3ω6, 20:2ω6, 20:3ω6, 20:4ω6, 22:5ω6, Σω6, ω6:ω3, and ΣPUFA (*p* = 0.0001–0.028). The transcript *acox1* showed negative correlations with 18:3ω6 and 22:5ω6 (*p* = 0.011 and 0.016, respectively; Figure 3).

#### Correlations Between Lipid Composition and Eicosanoid Metabolism Transcripts

The transcript expression of *ptges3* was correlated positively with 18:4ω3, 20:4ω3 and 18:0 (*p* = 0.012–0.049; Figure 3), while *ptges2* showed a negative correlation with 20:5ω3 (*p* = 0.041). The transcript expressions of *5loxa* and *5loxb* showed positive correlations with 20:5ω3 and 22:6ω3 (*p* = 0.002-0.045). *5loxb* also correlated positively with 20:4ω3 and Σω3 (*p* = 0.042 and 0.017, respectively), and negatively with ω6:ω3 (*p* = 0.044), while *5loxa* correlated positively with ST and 20:4ω6 (*p* = 0.012 and 0.006, respectively), and negatively with 18:4ω3, TAG, 18:1ω9 and ΣMUFA (*p* = 0.006-0.038; Figure 3). The mRNA levels of *cox1* showed positive correlations with the ω3 FA: 18:3ω3, 18:4ω3, 20:3ω3, 20:4ω3 and Σω3 (*p* = 0.009–0.043). The transcript *cox2* correlated positively with 16:0, ΣSFA and 22:4ω6 (*p* = 0.007–0.029), and negatively with 18:3ω6, 22:5ω6 and ΣPUFA (*p* = 0.013–0.037). Finally, *pgds* correlated negatively with ΣPUFA (*p* = 0.045; Figure 3).

## Discussion

### Head Kidney Lipid Composition

The head kidney was mainly composed of TAG (46.6–52.0%), and this was followed by the lipid classes PL (22.8–28.6%), and ST (18.4–23.7%) (Table 2). Our lipid class (i.e., TAG and ST) proportions are in the range of what was found in earlier studies on Atlantic salmon head kidney (Martinez-Rubio et al., 2013; Foroutani et al., 2020). Similar to our findings, Foroutani et al. (2020) reported that TAG was the predominant lipid class in Atlantic salmon head kidney. Head kidney is a principal component of the immune system and the major site of haematopoiesis in fish (Tort et al., 2003; Zapata et al., 2006). Therefore, high levels of TAG may allow fish to fulfill the energy required to fuel cell differentiation processes and other immune functions. Interestingly, Thompson et al. (1995) reported that TAG proportions in Atlantic salmon head kidney leucocytes, macrophages, T- and B-cells varied between ~14 and 20%, and indicated that TAG may serve as an energy store and/or a source of free fatty acids that are used in cellular functions in salmon. Our study also showed that lipid class composition [when expressed as % or as concentration (mg g^−1^ wet weight)] was not significantly affected by diet (Table 2, Supplementary Table 7). This result is in agreement with Betancor at al. (2014) who reported that dietary variation in LC-PUFA did not impact head kidney lipid classes in Atlantic salmon. However, the current study revealed that PL proportions were correlated positively with 20:5ω3 and negatively with 18:2ω6, 20:2ω6, and ω6:ω3 in the head kidney (Supplementary Table 8). Further, PCoA indicated associations between PL and the ω3 PUFA 22:6ω3, 20:5ω3, and Σω3 (Figure 1). These data suggest a preferential incorporation into the membrane of ω3 over ω6 PUFA. This can potentially increase the availability of anti-inflammatory eicosanoids in the head kidney (Martinez-Rubio et al., 2013). However, this can only be postulated, as fatty acid composition of the PL portion of the head kidney was not determined in our study.

The fatty acid composition of the head kidney reflected that of the experimental diets. For example, LC-PUFA precursor composition (i.e., 18:2ω6 and 18:3ω3) and ω6:ω3 ratio of the head kidney reflected the dietary composition (i.e., highest 18:2ω6 and ω6:ω3 in the high ω6 fed fish; highest 18:3ω3 in the high ω3 fed fish). Previous studies also indicated that head kidney lipid composition of Atlantic salmon (Gjøen et al., 2004; Foroutani et al., 2020), gilthead seabream (*Sparus aurata*) (Montero et al., 2003) and Nile tilapia (*Oreochromis niloticus*) (Chen et al., 2016) reflected dietary variation in 18:2ω6 and 18:3ω3. Gjøen et al. (2004) demonstrated that dietary replacement of FO with soy oil (i.e., 50%) modified head kidney ω6:ω3 (from 0.3 to 1.1), which reflected the dietary ω6:ω3. These ratios are comparable to the ω6:ω3 detected in our study (i.e., 0.5–1.5). At week 12, the 0.3% EPA+DHA↑ω6 fed fish had 1.7–2.3-fold more 20:4ω6 in terms of proportions and 1.4–1.8-fold in terms of concentrations, when compared with the other treatments. However, 20:5ω3 proportions (0.6–0.7-fold) and concentrations (0.4–0.6-fold) were lowest in this group, compared to the other treatments. Additionally, both proportions and concentrations of 20:5ω3 were similar between the 0.3% EPA+DHA↑ω3 and the 1% EPA+DHA↑ω3 fed fish. A similar effect on head kidney 20:4ω6 proportions was shown in a previous study where soy oil fed Atlantic salmon had 3-fold higher 20:4ω6 than FO fed fish (Gjøen et al., 2004). Furthermore, the differences in 20:4ω6 and 20:5ω3 proportions are in line with our previous study with Atlantic salmon muscle (Emam et al., 2020). Taken together, these data highlight the importance of dietary LC-PUFA precursors (i.e., 18:2ω6 and 18:3ω3) when salmon are fed with lower inclusion of EPA and DHA (0.3%), and their impact on head kidney LC-PUFA. Although LC-PUFA synthesis was not quantified in our study, the fact that intermediate ω6 and ω3 PUFAs were also significantly different among dietary treatments, and their head kidney proportions were higher than those of the diet, suggest that the LC-PUFA synthesis pathway was activated. Interestingly, 22:6ω3 proportions were not affected by diet at week 12, and 22:6ω3 concentrations (within the high ω3 and high ω6 treatments) did not vary with increasing dietary EPA+DHA levels. This suggests that in addition to elongation and desaturation, selective retention had also occurred. Indeed, earlier studies indicated higher 22:6ω3 retention in Atlantic salmon with decreasing dietary EPA+DHA (Glencross et al., 2014; Bou et al., 2017). However, the fact that 1% EPA+DHA/high↑ω3 and 1.4% EPA+DHA/balanced treatments showed higher concentration of 22:6ω3 than the 0.3% EPA+DHA/high↑ω6 fed fish suggests that both dietary EPA+DHA and ω6:ω3 influenced head kidney DHA content.

Salmon fed the 1.4% EPA+DHA/balanced diet had the highest level of ΣMUFA, and the 1% EPA+DHA↑ω3 fed fish showed higher ΣMUFA than the 0.3% EPA+DHA↑ω6 fed fish. These differences in ΣMUFA, and the observed changes in individual MUFA (i.e., 16:1ω7, 18:1ω7, 18:1ω9 and 20:1ω9) were also a reflection of the diets. Notably, the 1.4% EPA+DHA/balanced diet contained rapeseed oil which is rich in 18:1ω9 (Orsavova et al., 2015), and this may have influenced the observed changes in tissue MUFA. Emam et al. (2020) reported a similar response in salmon muscle, and showed that hepatic MUFA synthesis-related transcript *scdb* was positively correlated with muscle 20:1ω7 and 20:1ω9, among other MUFA. This suggests that head kidney MUFA differences between treatments in our study were also related to changes in MUFA synthesis. However, further analyses are required in order to test this hypothesis (e.g., transcript expression of MUFA synthesis-related genes in the head kidney).

### Transcript Expression of Lipid Metabolism-Related Genes

#### Transcripts Involved in LC-PUFA Synthesis

Transcript expression levels of key genes encoding enzymes involved in LC-PUFA synthesis were modulated by diet in our study. Salmon fed with 0.3% EPA+DHA↑ω6 had the lowest expression of *elovl5a*, while the remaining four treatments had similar levels of this transcript (Figure 2B and Supplementary Table 5). The transcript expression of other genes encoding elongases (*elovl4b*) and desaturases (*fadsd5, fadsd6b* and *fadsd6c)* was also similar among 0.3% EPA+DHA↑ω3, 1% EPA+DHA (both high ω3 and high ω6) and 1.4% EPA+DHA/balanced fed fish (Supplementary Table 5). Further, *elovl5a* was correlated positively with ω3 (20:3ω3 and 20:4ω3), while both *elovl5a* and *fadsd5* were correlated negatively with ω6 PUFA (18:2ω6, 18:3ω6, 20:2ω6, 20:3ω6, 20:4ω6, 22:5ω6) in the head kidney. These transcript expression changes along with the fatty acid data suggest that 20:5ω3 synthesis was lower in fish fed lower dietary levels of 18:3ω3 and EPA+DHA (i.e., 0.3%), and further support the notion that higher dietary 18:3ω3 promoted the synthesis of ω3 LC-PUFA in the head kidney. Indeed, LC-PUFA synthesis in salmonids is influenced by the availability of tissue LC-PUFA (i.e., EPA, DHA, ARA) and the precursors 18:3ω3 and 18:2ω6 (Jordal et al., 2005; Jump et al., 2005; Glencross et al., 2015; Katan et al., 2019). *Fadsd5* expression was higher with 1% EPA+DHA↑ω3 compared with 0.3% EPA+DHA↑ω6 fed fish, while *srebp1* showed higher expression levels in the 1% EPA+DHA↑ω3 and 1.4% EPA+DHA/balanced treatments compared with the 0.3% EPA+DHA↑ω6 fed fish. These transcripts, as well as *srebp2* and *lxrb*, were significantly positively impacted by dietary EPA+DHA levels (Figures 2C,D,I). Our results are in contrast with previous studies reporting up-regulation of hepatic *fadsd5* in Atlantic salmon fed plant-based (containing lower EPA and DHA) compared to FO diets (Zheng et al., 2004; Morais et al., 2011; Xue et al., 2015). However, our data is in agreement with Betancor et al. (2014) who reported up-regulation of head kidney *srebp1* and a ~1.5-fold increase in *fadsd5* in Atlantic salmon fed higher dietary levels of EPA+DHA (2% compared to 1% of diet). FO diets used in the former studies contained higher proportions of EPA+DHA (~11–24% of total FA) and higher inclusions of FO (~14–30% of diet) compared to our study (EPA+DHA proportions and FO inclusions were 2.8–8.8% and 0.1–6.8%, respectively). These as well as organ (i.e., liver vs. head kidney) differences may explain the discrepancies between studies. Further, dietary ratios of ALA:LNA were shown to influence LC-PUFA accumulation in salmonids and African catfish muscle tissues (Colombo et al., 2018; Sourabié et al., 2019), and this may have played a role in the regulation of LC-PUFA synthesis in our study. Finally, the positive impact of dietary EPA+DHA levels in our study could be related to MUFA levels, as the transcript expression of several lipid metabolism genes (*elovl5a, fadsd5*, and *srebp1*) showed a positive correlation with head kidney ΣMUFA (Figure 3). Interestingly, Emam et al. (2020) showed that hepatic *elovl5a* and *srebp2* were positively correlated with the levels of individual MUFA in salmon muscle. Indeed, an earlier study with Atlantic salmon revealed that LC-PUFA synthesis is stimulated by dietary MUFA levels, and suggested that the sparing effect of SFA and MUFA on ω3 LC-PUFA could be the underlying mechanism (Emery et al., 2016).

The transcript expression of head kidney *pparaa* was higher in the 0.3% EPA+DHA↑ω6 compared with the 0.3% EPA+DHA↑ω3 fed fish (Figure 2G), and correlation analysis indicated that this transcript was positively correlated with ω6 PUFA (18:2ω6, 20:2ω6, 20:3ω6, 20:4ω6, 22:5ω6), Σω6 and ω6:ω3, and negatively with ω3 PUFA (18:3ω3, 18:4ω3, 20:3ω3, 20:4ω3) and Σω3 (Figure 3). Ppara is a transcription factor that regulates numerous genes involved in lipid-related processes such as FA oxidation, as well as bile acid and triacylglycerol metabolism (Kersten, 2014). Further, several studies in fish demonstrated involvement of Ppara in LC-PUFA synthesis (Dong et al., 2017; Zhu et al., 2019). Thus, our data suggest that *pparaa* may have played a regulatory role in ω6 PUFA synthesis, particularly in the 0.3% EPA+DHA↑ω6 fed fish. Emam et al. (2020) showed that hepatic *pparaa* expression was higher in the 0.3% EPA+DHA↑ω3 than the 0.3% EPA+DHA↑ω6 and 1% EPA+DHA treatments (i.e., high ω3 and high ω6), and also a positive correlation between liver *pparaa* and muscle ω3 PUFA. These contrasting results suggest that *pparaa* response in Atlantic salmon is tissue dependent. Finally, the fact that the transcript expression of *pparaa* was significantly impacted by dietary ω6:ω3 (Figure 2I), and the correlation with head kidney ω6:ω3 (Figure 3), suggest that this transcript may have been involved with inflammatory processes in our study. This is in line with previous studies with mammals (Jones et al., 2002; Becker et al., 2008; Varga et al., 2011) and Atlantic salmon (Martinez-Rubio et al., 2013) which showed that Ppara and *ppara* play a role in the resolution of inflammation. More studies are required in order to investigate the dietary-induced impacts of *ppara* on inflammatory processes in Atlantic salmon head kidney.

#### Transcripts Involved in Eicosanoid Metabolism

Our qPCR results (Supplementary Table 5) are in line with previous studies which indicated no differences in head kidney constitutive transcript expression of several genes related to eicosanoid synthesis (i.e., *5lox, cox2*) in fish exposed to varying EPA levels [i.e., 0–20 μM (Furne et al., 2013), 1.1 and 19 mg g^−1^ lipid (Salini et al., 2016)] or ω6:ω3 ratios (0.7–4.1) (Holen et al., 2018). However, Holen et al. (2018) reported that Atlantic salmon fed with soybean oil as a FO replacement had higher expression of *pgds* and *pges* in head kidney leucocytes compared to fish fed palm and rapeseed oils. Differences in dietary and tissue EPA:ARA, EPA+DHA, the tissues/cells examined (head kidney *vs*. isolated leucocytes), and variation between *in vivo* and *in vitro* models could have contributed to these discrepancies between studies. Further, our study revealed that head kidney transcript expression of *cox1* was negatively impacted by dietary ω6:ω3 levels (Figures 2H,I), and positively correlated with head kidney ω3 fatty acids (i.e., 18:3ω3, 18:4ω3, 20:3ω3, 20:4ω3, and Σω3; Figure 3). The enzyme COX1 is constitutively expressed in several tissues (e.g., liver, kidney, spleen, gill, muscle, gut) and has maintenance and homeostatic functions (Tapiero et al., 2002; Olsen et al., 2012). Our data suggest that *cox1* expression could result in production of anti-inflammatory prostaglandins in Atlantic salmon head kidney at the constitutive level. Further, differences among treatments in head kidney EPA:ARA (Table 2) may have altered the production of eicosanoids via *cox1* transcription. Indeed, changes in cell membrane EPA:ARA could alter eicosanoid production and the inflammatory response in vertebrates (Calder, 2003; Arts and Kohler, 2009; Salini et al., 2016). However, since eicosanoid levels were not measured in our study, these hypotheses could not be tested. Further, the implications of FA-transcript correlations on eicosanoid production could not be assessed.

In addition to *cox1*, transcript expression of *5loxa, 5loxb*, and *ptges3* was also positively correlated with head kidney ω3 LC-PUFA. However, *5loxa* showed positive correlations with both ω3 (i.e., 20:5ω3, 22:6ω3) and ω6 (20:4ω6) LC-PUFA. The latter result is in line with Katan et al. (2019) who reported that hepatic *5loxa* showed a positive relationship (*p* = 0.059) with liver EPA+ARA in Atlantic salmon fed different mixes of plant oils as a FO replacement. However, this is in contrast with Caballero-Solares et al. (2020) who showed that hepatic *5loxa* correlated negatively with liver EPA and DHA in salmon fed terrestrial plant ingredients as FO and FM alternatives. Discrepancies between studies suggest that the interaction of *5loxa* transcription with tissue LC-PUFA in salmon depends on tissue (i.e., head kidney vs. liver) and/or dietary inputs (e.g., protein and lipid sources). Our study also showed positive correlations between ω3 PUFA (i.e., 18:4ω3, 20:4ω3) and *ptges3* expression, and negative correlations between EPA and *ptges2*. In mammals, both PTGES2 and PTGES3 convert PGH_2_ into PGE_2_ (Neuman et al., 2017; Xu et al., 2019). Wei et al. (2016) showed that muscle *ptges3* expression was induced in pigs fed with linseed-enriched diets for 60 days. However, previous studies reported no impacts of dietary fatty acids on the transcript expression of *ptges2* and *ptges3* in bovine oocytes (Ponter et al., 2012) and Eurasian perch (*Perca fluviatilis*) liver, brain and intestine (Geay et al., 2015). Clearly, more research is required to elucidate the influence of dietary and tissue PUFA on the transcript expression of *ptges2* and *ptges3* in fish.

*Cox2* showed a positive correlation with 16:0, ΣSFA and 22:4ω6, and negative correlations with ΣPUFA, 18:3ω6 and 22:5ω6 in the current study (Figure 3). This finding supports previous human macrophage studies which indicated that SFA induced *cox2* expression (Lee et al., 2001; Rocha et al., 2016). Astiz et al. (2012) showed that addition of 22:4ω6 and 22:5ω6 did not impact the production of COX2 in rat testis cells. However, a previous study on mouse brain indicated that reduction in the levels of ARA metabolites 22:4ω6 and 22:5ω6 reflected a shift of ARA from LC-PUFA biosynthesis prostaglandin or leukotriene synthesis (McNamara et al., 2009). Thus, the negative correlation between 22:5ω6 and *cox2* could be a result of higher availability of ARA for COX-2 mediated prostaglandin synthesis. Finally, similarly to *cox2, pgds* correlated negatively with ΣPUFA. This latter finding is in line with Katan et al. (2019) who showed that hepatic *pgds* was negatively correlated with liver EPA+ARA+DHA in Atlantic salmon. However, Holen et al. (2015) reported that the combination of EPA+ARA+DHA did not modulate *cox2* transcript expression in Atlantic salmon head kidney cells. In summary, further examination is needed in order to elucidate the influence of ARA metabolites (i.e., 22:4ω6 and 22:5ω6) on *cox2* expression, and the interactive impacts of PUFA on the transcript expression of *pgds* and *cox2* in Atlantic salmon head kidney.

## Conclusion

This study investigated head kidney lipid composition and transcript expression of genes involved in fatty acid and eicosanoid metabolism in Atlantic salmon fed varying dietary levels of EPA+DHA (0.3, 1.0, and 1.4%) and ω6:ω3 ratios (high ω6, high ω3, and balanced) for 12 weeks. TAG was the predominant lipid class in all treatments, regardless of diet. This suggests that this lipid class could play an important role in immune and/differentiation processes in Atlantic salmon head kidney. Head kidney fatty acid composition was reflective of the diet with respect to C_18_ PUFA and MUFA levels (% of total fatty acids), and responded to ω6:ω3 variation. Proportions of 20:5ω3 were similar among 0.3% EPA+DHA↑ω3, 1% EPA+DHA (both high ω3 and high ω6 treatments) and 1.4% EPA+DHA/balanced fed fish, although dietary 20:5ω3 varied by 2.5- to 3-fold. Tissue fatty acid composition changes agreed with positive correlations between head kidney 20:3ω3 and 20:4ω3 and *elovl5a* transcript levels. This suggested that high dietary 18:3ω3 promoted the synthesis of ω3 LC-PUFA in salmon fed lower dietary EPA+DHA levels (0.3%). Further, head kidney transcript expression of several genes involved in FA metabolism (e.g., *elovl5a, fadsd5* and *srebp1)* was positively impacted by dietary EPA+DHA levels, and showed a positive correlation with head kidney ΣMUFA. This supported the hypothesis that LC-PUFA synthesis is stimulated by increasing MUFA levels in Atlantic salmon.

Several eicosanoid synthesis-related transcripts were significantly correlated with head kidney fatty acid composition. The head kidney transcript expression of *5loxb* correlated positively with ω3 LC-PUFA and Σω3, and correlated negatively with ω6:ω3. *Cox1* and *ptges3* transcript expression correlated positively with ω3 PUFA, while *cox1* was negatively impacted by dietary ω6:ω3 levels. We hypothesize that these transcript expression changes may have increased the production of anti-inflammatory eicosanoids in salmon head kidney. Finally, our study revealed significant positive and negative correlations of *cox2* expression with SFA (i.e., 16:0, ΣSFA) and 22:5ω6, respectively. Given the important role of the head kidney in salmon immune responses and the interaction with dietary LC-PUFA, future studies should elucidate how ω6:ω3 and EPA+DHA levels affect LC-PUFA synthesis (e.g., stable isotope and FA mass balance methods) and eicosanoid production (e.g., LC-MS/MS) in this organ.

## Data Availability Statement

The raw data supporting the conclusions of this article will be made available by the authors, without undue reservation.

## Ethics Statement

The animal study was reviewed and approved by Animal Care Committee of Memorial University of Newfoundland.

## Author Contributions

MR, CP, and RT contributed to conception and design of the study. TK was involved in all aspects of the study, including experimental design, data and statistical analyses, and writing of the first manuscript draft. TK, XX, and AC-S took part in sampling. XX assisted with analyses related to the qPCR study. AC-S assisted with the Pearson correlation matrix. All authors contributed to original manuscript editing, revisions, and they read and approved the submitted version.

## Conflict of Interest

RT was employed by the company Cargill, but did not have any additional role in the study design, data collection, analysis and interpretation, writing of the manuscript, or in the decision to submit the manuscript for publication. The remaining authors declare that the research was conducted in the absence of any commercial or financial relationships that could be construed as a potential conflict of interest.
