# Supplementary material for: Influence of Dietary Long-Chain Polyunsaturated Fatty Acids and ω6 to ω3 Ratios on Head Kidney Lipid Composition and Expression of Fatty Acid and Eicosanoid Metabolism Genes in Atlantic Salmon (Salmo salar)
Source: Front Mol Biosci. 2020 Dec 14;7:602587. doi: 10.3389/fmolb.2020.602587 (PMC7767880; doi:10.3389/fmolb.2020.602587)
Supplement: Supplementary file 1 [file Table_1.docx]

**Supplementary Material**

**Table S1.** Formulation and nutrient composition (%) of experimental diets fed to Atlantic salmon for 12 weeks. Modified from Emam et al. (2020).

| Ingredient (% of diet)^a^ | 0.3%EPA+DHA | 0.3%EPA+DHA | 1%EPA+DHA | 1%EPA+DHA | 1.4%EPA+DHA |
| --- | --- | --- | --- | --- | --- |
|  | ↑ω6 | ↑ω3 | ↑ω6 | ↑ω3 | balanced |
| Fish oil | 0.1 | - | 4.3 | 4.3 | 6.8 |
| Soy oil | 12.5 | - | 10.1 | - | - |
| Linseed oil | - | 7.9 | - | 6.5 | - |
| Poultry fat^b^ | 2.4 | 7.1 | 0.6 | 4.3 | 3.4 |
| Rapeseed oil | - | - | - | - | 4.8 |
| Proximate composition (% as fed basis) |  |  |  |  |  |
| Nitrogen^c^ | 7.3 | 7.5 | 7.8 | 7.3 | 7.2 |
| Crude lipid^d^ | 19.9 | 20.6 | 20.0 | 20.7 | 20.4 |
| Dry matter^d^ | 97.4 | 97.6 | 98.0 | 97.5 | 97.8 |
| Ash^d^ | 5.7 | 5.7 | 5.4 | 5.7 | 6.0 |

^a^ All ingredients were sourced from EWOS stocks.

^b^ Poultry fat contained EPA+DHA and 18:2ω6 levels of 0.4 and 2.9% of total FA, respectively. This contributed to their level in experimental diets.

^c^ Analysed as % of dry weight (n=3).

^d^ Analysed as % of wet weight (n=3).

**Table S2.** Lipid and fatty acid composition (%) of experimental diets^a^ fed to Atlantic salmon for 12 weeks. Modified from Emam et al. (2020).

|  | 0.3%EPA+DHA | 0.3%EPA+DHA | 1%EPA+DHA | 1%EPA+DHA | 1.4%EPA+DHA |
| --- | --- | --- | --- | --- | --- |
|  | ↑ω6 | ↑ω3 | ↑ω6 | ↑ω3 | balanced |
| Lipid classes composition (% of total lipid) | |  |  |  |  |
| TAG (%)^b^ | 62.2 | 68.4 | 70.6 | 60.9 | 71.6 |
| ST (%)^c^ | 3.8 | 5.5 | 3.8 | 3.4 | 3.4 |
| AMPL (%)^d^ | 2.1 | 2.7 | 1.8 | 1.7 | 2.1 |
| PL (%)^e^ | 11.7 | 6.0 | 4.4 | 10.2 | 6.3 |
| TL (mg/g)^f^ | 174.0 | 189.1 | 168.9 | 210.1 | 201.8 |
| Fatty acid composition (% of total FA) | |  |  |  |  |
| 14:0 | 0.7 | 0.8 | 1.9 | 2.0 | 2.7 |
| 16:0 | 14.0 | 14.0 | 13.5 | 13.3 | 12.9 |
| 16:1ω7 | 1.2 | 2.3 | 1.8 | 2.7 | 3.2 |
| 18:0 | 5.2 | 5.8 | 4.7 | 5.0 | 4.0 |
| 18:1ω7 | 1.7 | 1.6 | 1.9 | 2.0 | 2.9 |
| 18:1ω9 | 23.2 | 28.1 | 18.6 | 22.5 | 29.5 |
| **18:2ω6 (LNA)** | **41.4** | **18.3** | **33.4** | **14.8** | **13.3** |
| **18:3ω3 (ALA)** | **6.0** | **22.4** | **5.0** | **18.4** | **4.1** |
| 20:1ω9 | 0.3 | 0.4 | 1.0 | 1.0 | 1.7 |
| 20:1ω11 | 0.1 | 0.1 | 2.5 | 2.6 | 4.1 |
| 20:4ω6 (ARA) | 0.3 | 0.4 | 0.3 | 0.4 | 0.4 |
| **20:5ω3 (EPA)** | **1.1** | **1.2** | **3.1** | **3.1** | **4.3** |
| 22:1ω11 | 0.2 | 0.1 | 2.6 | 2.7 | 4.2 |
| **22:6ω3 (DHA)** | **1.7** | **1.8** | **3.6** | **3.5** | **4.5** |
| ΣSFA^g^ | 21.1 | 21.4 | 21.4 | 21.3 | 20.9 |
| ΣMUFA^h^ | 27.2 | 33.2 | 29.6 | 34.8 | 47.3 |
| ΣPUFA^i^ | 51.6 | 45.2 | 48.8 | 43.6 | 31.4 |
| **Σω3** | **9.4** | **25.9** | **14.1** | **27.4** | **16.4** |
| **Σω6** | **41.9** | **19.0** | **34.0** | **15.6** | **14.2** |
| ω6:ω3 | 4.5 | 0.7 | 2.4 | 0.6 | 0.9 |
| EPA+DHA | 2.8 | 3.0 | 6.7 | 6.6 | 8.8 |
| DHA:EPA | 1.5 | 1.5 | 1.2 | 1.1 | 1.0 |
| EPA:ARA | 3.7 | 3.0 | 10.3 | 7.8 | 10.8 |

^a^ Mean (n=3). ^b^ Triacylglycerol. ^c^ Sterols. ^d^ Acetone mobile polar lipid. ^e^ Phospholipids. ^f^ Total lipids. ^g^ Total saturated fatty acids. ^h^ Total monounsaturated fatty acids. ^i^ Total polyunsaturated fatty acids. Fatty acids in bold font were key to the experimental design.

**Table S3.** PERMANOVA pairwise test^a^ of head kidney FA and lipid composition compared across dietary treatments.

| Dietary treatments | t | P(perm)^b^ |
| --- | --- | --- |
| 0.3%EPA+DHA↑ω6, 0.3%EPA+DHA↑ω3 | 5.11 | **0.0001** |
| 0.3%EPA+DHA↑ω6, 1%EPA+DHA↑ω6 | 2.40 | **0.003** |
| 0.3%EPA+DHA↑ω6, 1%EPA+DHA↑ω3 | 5.64 | **0.0001** |
| 0.3%EPA+DHA↑ω6, 1.4%EPA+DHA/balanced | 5.91 | **0.0001** |
| 0.3%EPA+DHA↑ω3, 1%EPA+DHA↑ω6 | 3.64 | **0.0001** |
| 0.3%EPA+DHA↑ω3, 1%EPA+DHA↑ω3 | 1.50 | 0.12 |
| 0.3%EPA+DHA↑ω3, 1.4%EPA+DHA/balanced | 3.13 | **0.0002** |
| 1%EPA+DHA↑ω6, 1%EPA+DHA↑ω3 | 3.76 | **0.0001** |
| 1%EPA+DHA↑ω6, 1.4%EPA+DHA/balanced | 4.19 | **0.0001** |
| 1%EPA+DHA↑ω3, 1.4%EPA+DHA/balanced | 2.84 | **0.002** |

Significant P(perm)-values (i.e. p < 0.05) are in bold font.

^a^ The non-parametric Bray-Curtis similarity was used with 9999 permutations

(see Materials and Methods).

^b^ PERMANOVA p-values for all dietary treatment comparisons.

**Table S4**. SIMPER dissimilarities of head kidney FA and lipid composition compared across dietary treatments.

| Dietary treatments | Average dissimilarity (%) | Major lipid class / FA contributor^a^ | Contribution (%) |
| --- | --- | --- | --- |
| 0.3%EPA+DHA↑ω6 & | 15.6 | TAG | 12.9 |
| 0.3%EPA+DHA↑ω3 |  | PL | 12.6 |
|  |  | Σω6 | 12.1 |
|  |  | Σω3 | 7.7 |
|  |  | ST | 7.4 |
| 0.3%EPA+DHA↑ω6 & | 11.0 | TAG | 15.6 |
| 1%EPA+DHA↑ω6 |  | PL | 15.6 |
|  |  | ST | 9.2 |
|  |  | Σω6 | 7.7 |
|  |  | Σω3 | 6.2 |
| 0.3%EPA+DHA↑ω3 & | 12.5 | TAG | 15.0 |
| 1%EPA+DHA↑ω6 |  | PL | 13.8 |
|  |  | ST | 9.2 |
|  |  | Σω6 | 8.5 |
|  |  | 18:2ω6 | 6.8 |
| 0.3%EPA+DHA↑ω6 & | 16.6 | Σω6 | 13.1 |
| 1%EPA+DHA↑ω3 |  | TAG | 12.2 |
|  |  | PL | 11.2 |
|  |  | Σω3 | 8.8 |
|  |  | 18:2ω6 | 7.9 |
| 0.3%EPA+DHA↑ω3 & | 10.0 | TAG | 22.1 |
| 1%EPA+DHA↑ω3 |  | PL | 17.9 |
|  |  | ST | 10.9 |
| 1%EPA+DHA↑ω6 & | 12.7 | TAG | 14.7 |
| 1%EPA+DHA↑ω3 |  | PL | 12.4 |
|  |  | Σω6 | 10.6 |
|  |  | ST | 8.0 |
|  |  | 18:2ω6 | 7.9 |
| 0.3%EPA+DHA↑ω6 & | 18.7 | Σω6 | 12.4 |
| 1.4%EPA+DHA/balanced |  | TAG | 12.1 |
|  |  | PL | 10.4 |
|  |  | ΣMUFA | 9.2 |
|  |  | ΣPUFA | 9.2 |
| 0.3%EPA+DHA↑ω3 & | 12.7 | TAG | 18.3 |
| 1.4%EPA+DHA/balanced |  | PL | 14.8 |
|  |  | ST | 9.2 |
|  |  | ΣPUFA | 7.8 |
| 1%EPA+DHA↑ω6 & | 14.9 | TAG | 14.3 |
| 1.4%EPA+DHA/balanced |  | PL | 11.0 |
|  |  | Σω6 | 10.0 |
|  |  | ΣMUFA | 9.7 |
|  |  | ΣPUFA | 9.7 |
| 1%EPA+DHA↑ω3 & | 12.3 | TAG | 19.6 |
| 1.4%EPA+DHA/balanced |  | PL | 14.5 |
|  |  | ST | 9.2 |
|  |  | ΣPUFA | 8.0 |

^a^ TAG, PL and ST represent triacylglycerol, phospholipids, and sterols, respectively. ΣMUFA and ΣPUFA represent total monounsaturated and polyunsaturated fatty acids, respectively.

**Table S5.** Relative transcript expression of genes related to FA and eicosanoid metabolism in the head kidney of Atlantic salmon after 12 weeks of feeding diets with different ratios of ω6:ω3 and levels of EPA+DHA.

| Transcript^a^ | 0.3%EPA+DHA | 0.3%EPA+DHA | 1%EPA+DHA | 1%EPA+DHA | 1.4%EPA+DHA | p-value^b^ |
| --- | --- | --- | --- | --- | --- | --- |
|  | ↑ω6 | ↑ω3 | ↑ω6 | ↑ω3 | balanced |  |
| *elovl4b* | 1.6 ± 0.4 | 1.5 ± 0.3 | 1.5 ± 0.4 | 1.9 ± 0.3 | 1.6 ± 0.4 | 0.24 |
| *elovl5a* | 1.5 ± 0.4^a^ | 2.0 ± 0.3^b^ | 2.0 ± 0.3^b^ | 2.1 ± 0.3^b^ | 2.0 ± 0.1^b^ | **0.01** |
| *fadsd5* | 2.0 ± 0.5^a^ | 2.6 ± 0.9^ab^ | 3.2 ± 1.2^ab^ | 3.3 ± 0.7^b^ | 3.0 ± 0.5^ab^ | **0.04** |
| *fadsd6b* | 3.1 ± 1.3 | 3.2 ± 1.6 | 2.7 ± 0.8 | 2.8 ± 0.3 | 2.9 ± 0.7 | 0.70 |
| *fadsd6c* | 3.0 ± 1.2 | 2.9 ± 1.1 | 3.1 ± 1.3 | 3.9 ± 1.3 | 3.5 ± 1.1 | 0.50 |
| *srebp1* | 1.6 ± 0.4^a^ | 1.8 ± 0.6^ab^ | 2.0 ± 0.3^ab^ | 2.4 ± 0.3^b^ | 2.3 ± 0.5^b^ | **0.02** |
| *srebp2* | 1.7 ± 0.6 | 1.8 ± 0.4 | 2.2 ± 0.5 | 2.3 ± 0.6 | 2.0 ± 0.4 | 0.11 |
| *lxra* | 1.5 ± 0.3 | 1.5 ± 0.3 | 1.5 ± 0.2 | 1.7 ± 0.2 | 1.8 ± 0.3 | 0.23 |
| *lxrb* | 2.3 ± 0.4 | 2.4 ± 0.8 | 2.8 ± 0.9 | 3.0 ± 0.7 | 2.4 ± 0.6 | 0.35 |
| *pparaa* | 7.4 ± 4.5^a^ | 2.8 ± 1.4^b^ | 5.1 ± 2.0^ab^ | 3.8 ± 1.9^ab^ | 6.2 ± 3.0^ab^ | **0.02** |
| *pparb1* | 2.1 ± 0.5 | 2.0 ± 0.2 | 1.9 ± 0.6 | 2.0 ± 0.4 | 1.8 ± 0.3 | 0.68 |
| *pparb2a* | 1.4 ± 0.3 | 1.5 ± 0.3 | 1.5 ± 0.3 | 1.6 ± 0.3 | 1.4 ± 0.3 | 0.33 |
| *fasa* | 2.0 ± 0.9 | 2.0 ± 0.7 | 2.0 ± 0.6 | 2.3 ± 0.6 | 1.9 ± 0.6 | 0.85 |
| *fasb* | 4.0 ± 1.9 | 3.6 ± 1.4 | 3.5 ± 0.9 | 3.7 ± 1.1 | 3.0 ± 0.8 | 0.78 |
| *acox1* | 1.5 ± 0.4 | 1.7 ± 0.4 | 1.7 ± 0.3 | 1.8 ± 0.3 | 1.6 ± 0.2 | 0.62 |
| *cpt1a* | 1.5 ± 0.3 | 1.5 ± 0.1 | 1.5 ± 0.3 | 1.4 ± 0.3 | 1.5 ± 0.2 | 0.93 |
| *cpla2* | 1.3 ± 0.2 | 1.3 ± 0.2 | 1.3 ± 0.2 | 1.3 ± 0.1 | 1.4 ± 0.2 | 0.97 |
| *cox1* | 1.6 ± 0.3 | 1.8 ± 0.4 | 1.5 ± 0.2 | 1.8 ± 0.5 | 1.5 ± 0.3 | 0.19 |
| *cox2* | 3.3 ± 2.1 | 3.3 ± 0.8 | 4.2 ± 1.5 | 3.1 ± 1.5 | 3.9 ± 1.7 | 0.58 |
| *5loxa* | 1.9 ± 0.2 | 1.7 ± 0.4 | 1.9 ± 0.5 | 1.7 ± 0.5 | 1.8 ± 0.6 | 0.88 |
| *5loxb* | 1.5 ± 0.3 | 1.7 ± 0.3 | 1.6 ± 0.4 | 1.7 ± 0.4 | 1.7 ± 0.5 | 0.82 |
| *pgds* | 2.6 ± 1.1 | 3.4 ± 1.7 | 3.0 ± 1.1 | 3.1 ± 0.4 | 2.8 ± 0.9 | 0.74 |
| *ptges2* | 2.1 ± 0.6 | 2.1 ± 0.5 | 2.0 ± 0.8 | 2.0 ± 0.6 | 1.6 ± 0.4 | 0.57 |
| *ptges3* | 1.6 ± 0.5 | 2.1 ± 0.2 | 1.9 ± 0.5 | 1.9 ± 0.4 | 1.6 ± 0.4 | 0.06 |
| *ikha4* | 1.6 ± 0.4 | 1.5 ± 0.2 | 1.7 ± 0.7 | 1.6 ± 0.2 | 1.4 ± 0.3 | 0.71 |
|  |  |  |  |  |  |  |

Significant p-values (i.e. p < 0.05) are in bold font.

^a^ Transcript expression values presented as mean relative quantity (RQ) ± SD (n = 7–8). RQs were normalized to eukaryotic translation initiation factor 3 subunit D (*eif3d*) and polyadenylate-binding protein cytoplasmic 1 (*pabpc1*), and calibrated to the lowest expressing individual for each gene of interest (see Materials and Methods).

^b^ Different letters indicate significant differences among treatments (one-way ANOVA followed by Tukey post-hoc tests; see Materials and Methods).

**Table S6**. Two-way ANOVA analysis^a^ illustrating effects of dietary EPA+DHA (i.e. 0.3 and 1%) and ω6:ω3 (i.e. high ω6 and high ω3) on the transcript expression of genes related to FA and eicosanoid metabolism in Atlantic salmon head kidney.

| Transcript | EPA+DHA | ω6:ω3 | Interaction |
| --- | --- | --- | --- |
| *elovl4b* | 0.34 | 0.22 | **0.04** |
| *elovl5a* | **0.03** | **0.01** | 0.11 |
| *fadsd5* | **0.01** | 0.27 | 0.48 |
| *fadsd6b* | 0.31 | 0.77 | 0.90 |
| *fadsd6c* | 0.24 | 0.43 | 0.27 |
| *srebp1* | **0.01** | 0.09 | 0.72 |
| *srebp2* | **0.01** | 0.58 | 0.93 |
| *lxra* | 0.28 | 0.31 | 0.39 |
| *lxrb* | **0.048** | 0.57 | 0.83 |
| *pparaa* | 0.50 | **0.004** | 0.09 |
| *pparb1* | 0.53 | 0.86 | 0.45 |
| *pparb2a* | 0.35 | 0.19 | 0.89 |
| *fasa* | 0.48 | 0.55 | 0.55 |
| *fasb* | 0.67 | 0.87 | 0.56 |
| *acox1* | 0.32 | 0.43 | 0.76 |
| *cpt1a* | 0.55 | 0.69 | 0.77 |
| *cpla2* | 0.88 | 0.68 | 0.99 |
| *cox1* | 0.90 | **0.03** | 0.63 |
| *cox2* | 0.53 | 0.35 | 0.35 |
| *5loxa* | 0.65 | 0.20 | 0.93 |
| *5loxb* | 0.70 | 0.30 | 0.92 |
| *pgds* | 0.85 | 0.31 | 0.40 |
| *ptges2* | 0.77 | 0.86 | 0.87 |
| *ptges3* | 0.65 | 0.14 | 0.09 |
| *ikha4* | 0.54 | 0.30 | 0.93 |

^a^ p-values corresponding to the factors EPA+DHA, ω6:ω3, and their interaction (see Materials and Methods). Significant p-values (i.e. p < 0.05) are in bold font.

**Table S7.** Lipid class and fatty acid composition (mg g^-1^ wet weight) of Atlantic salmon head kidney^a^ before (Initial) and after 12 weeks of feeding diets with different ratios of ω6:ω3 and levels of EPA+DHA.

|  | Initial | 0.3%EPA+DHA | 0.3%EPA+DHA | 1%EPA+DHA | 1%EPA+DHA | 1.4%EPA+DHA |
| --- | --- | --- | --- | --- | --- | --- |
|  |  | ↑ω6 | ↑ω3 | ↑ω6 | ↑ω3 | balanced |
| Lipid classes composition (mg g^-1^) | |  |  |  |  |  |
| TAG^b^ | 14.2 ± 10.2 | 12.9 ± 6.0 | 17.3 ± 8.1 | 15.7 ± 6.4 | 18.2 ± 14.9 | 20.3 ± 13.1 |
| ST^c^ | 9.9 ± 2.1 | 6.1 ± 1.7 | 6.5 ± 1.8 | 7.1 ± 1.5 | 7.1 ± 2.1 | 6.0 ± 1.5 |
| PL^d^ | 6.6 ± 2.9 | 7.2 ± 3.4 | 9.2 ± 3.7 | 7.0 ± 2.9 | 8.8 ± 2.8 | 8.5 ± 3.4 |
| Fatty acid composition (mg g^-1^) | |  |  |  |  |  |
| 14:0 | 0.4 ± 0.3 | 0.2 ± 0.1^a^ | 0.3 ± 0.1^ab^ | 0.3 ± 0.2^ab^ | 0.4 ± 0.3^bc^ | 0.5 ± 0.3^c^ |
| 16:0 | 2.7 ± 1.7 | 2.6 ± 0.8 | 3.3 ± 1.1 | 3.3 ± 1.4 | 3.2 ± 1.6 | 3.6 ± 1.7 |
| 16:1ω7 | 0.9 ± 0.6 | 0.4 ± 0.2^a^ | 0.7 ± 0.3^ab^ | 0.5 ± 0.3^ab^ | 0.8 ± 0.7^ab^ | 0.9 ± 0.6^b^ |
| 18:0 | 0.9 ± 0.7 | 1.0 ± 0.3 | 1.2 ± 0.4 | 1.2 ± 0.6 | 1.1 ± 0.5 | 1.1 ± 0.5 |
| 18:1ω9 | 5.1 ± 3.1 | 3.7 ± 1.6^a^ | 5.9 ± 2.4^ab^ | 4.3 ± 1.9^ab^ | 5.7 ± 4.0^ab^ | 7.0 ± 4.3^b^ |
| 18:1ω7 | 0.6 ± 0.4 | 0.4 ± 0.1^a^ | 0.5 ± 0.2^a^ | 0.5 ± 0.2^a^ | 0.6 ± 0.4^ab^ | 0.8 ± 0.4^b^ |
| 18:2ω6 (LNA) | 1.6 ± 1.3 | 3.5 ± 1.7 | 2.8 ± 1.1 | 3.3 ± 1.3 | 2.7 ± 1.9 | 2.6 ± 1.6 |
| 18:3ω6 | 0.1 ± 0.05 | 0.2 ± 0.1^a^ | 0.1 ± 0.1^ab^ | 0.1 ± 0.04^b^ | 0.1 ± 0.1^b^ | 0.1 ± 0.1^b^ |
| 18:3ω3 (ALA) | 0.2 ± 0.2 | 0.3 ± 0.2^a^ | 1.6 ± 0.7^b^ | 0.4 ± 0.2^a^ | 1.6 ± 1.0^b^ | 0.5 ± 0.3^a^ |
| 18:4ω3 | 0.1 ± 0.1 | 0.1 ± 0.1^a^ | 0.4 ± 0.2^c^ | 0.2 ± 0.1^a^ | 0.4 ± 0.2^bc^ | 0.3 ± 0.2^ab^ |
| 20:1ω11 | 0.1 ± 0.1 | 0.02 ± 0.01^a^ | 0.1 ± 0.1^a^ | 0.2 ± 0.1^b^ | 0.2 ± 0.2^b^ | 0.4 ± 0.2^c^ |
| 20:1ω9 | 0.4 ± 0.3 | 0.2 ± 0.1^a^ | 0.3 ± 0.2^a^ | 0.3 ± 0.2^ab^ | 0.4 ± 0.4^ab^ | 0.6 ± 0.4^b^ |
| 20:2ω6 | 0.1 ± 0.1 | 0.3 ± 0.1^a^ | 0.2 ± 0.1^b^ | 0.2 ± 0.1^a^ | 0.2 ± 0.1^ab^ | 0.2 ± 0.1^ab^ |
| 20:3ω6 (DGLA) | 0.1 ± 0.1 | 0.5 ± 0.1^a^ | 0.2 ± 0.1^bc^ | 0.3 ± 0.1^b^ | 0.2 ± 0.1^c^ | 0.2 ± 0.1^c^ |
| 20:4ω6 (ARA) | 0.2 ± 0.2 | 0.7 ± 0.2^a^ | 0.5 ± 0.1^b^ | 0.5 ± 0.1^b^ | 0.4 ± 0.1^b^ | 0.4 ± 0.1^b^ |
| 20:3ω3 | 0.01 ± 0.01 | 0.03 ± 0.01^a^ | 0.1 ± 0.04^b^ | 0.03 ± 0.01^a^ | 0.1 ± 0.1^b^ | 0.04 ± 0.02^a^ |
| 20:4ω3 | 0.1 ± 0.1 | 0.1 ± 0.03^a^ | 0.2 ± 0.1^b^ | 0.1 ± 0.1^a^ | 0.2 ± 0.1^b^ | 0.2 ± 0.1^b^ |
| 20:5ω3 (EPA) | 0.5 ± 0.5 | 0.4 ± 0.1^a^ | 0.8 ± 0.2^b^ | 0.7 ± 0.2^b^ | 1.0 ± 0.4^bc^ | 1.1 ± 0.4^c^ |
| 22:1ω11 | 0.4 ± 0.2 | 0.1 ± 0.1^a^ | 0.2 ± 0.1^ab^ | 0.3 ± 0.1^ab^ | 0.4 ± 0.3^bc^ | 0.5 ± 0.4^c^ |
| 22:4ω6 | 0.02 ± 0.03 | 0.04 ± 0.01 | 0.03 ± 0.01 | 0.03 ± 0.02 | 0.03 ± 0.01 | 0.03 ± 0.01 |
| 22:5ω6 (ω6 DPA) | 0.02 ± 0.02 | 0.1 ± 0.02^a^ | 0.1 ± 0.01^b^ | 0.04 ± 0.01^b^ | 0.04 ± 0.02^b^ | 0.05 ± 0.02^b^ |
| 22:5ω3 (ω3 DPA) | 0.2 ± 0.2 | 0.1 ± 0.1^a^ | 0.2 ± 0.1^ab^ | 0.2 ± 0.1^ab^ | 0.3 ± 0.2^b^ | 0.3 ± 0.2^b^ |
| 22:6ω3 (DHA) | 1.2 ± 1.1 | 2.1 ± 0.5^a^ | 2.8 ± 0.7^abc^ | 2.6 ± 0.7^ab^ | 3.1 ± 1.1^bc^ | 3.4 ± 0.9^c^ |
| ΣSFA^e^ | 4.3 ± 2.7 | 3.9 ± 1.2 | 4.9 ± 1.6 | 5.0 ± 2.2 | 4.9 ± 2.5 | 5.5 ± 2.6 |
| ΣMUFA^f^ | 8.5 ± 5.2 | 5.0 ± 2.2^a^ | 8.0 ± 3.3^ab^ | 6.6 ± 2.9^a^ | 8.5 ± 6.1^ab^ | 10.6 ± 6.5^b^ |
| ΣPUFA^g^ | 4.5 ± 3.9 | 8.7 ± 2.8 | 10.3 ± 3.3 | 9.0 ± 2.6 | 10.4 ± 5.2 | 9.7 ± 4.2 |
| Σω3 | 2.2 ± 2.1 | 3.3 ± 0.9^a^ | 6.2 ± 1.9^c^ | 4.3 ± 1.2^ab^ | 6.7 ± 2.8^c^ | 5.9 ± 2.1^bc^ |
| Σω6 | 2.0 ± 1.6 | 5.3 ± 2.0 | 3.9 ± 1.4 | 4.5 ± 1.5 | 3.6 ± 2.2 | 3.6 ± 2.0 |
| EPA+DHA | 2.0 ± 1.6 | 2.5 ± 0.6^a^ | 3.6 ± 0.9^bc^ | 3.4 ± 0.9^ab^ | 4.1 ± 1.5^bc^ | 4.5 ± 1.3^c^ |

^a^ Mean (n = 10-20) ± standard deviation (SD). Different superscripts in the same row indicate significant differences among treatments at week 12. Underlines represent values that are significantly different to week 0 (Initial) (p < 0.05). ^b^ Triacylglycerol. ^c^ Sterols. ^d^ Phospholipids. ^e^ Total saturated fatty acids. ^f^ Total monounsaturated fatty acids. ^g^ Total polyunsaturated fatty acids.

**Table S8**. Significant Pearson correlations^a^ (p < 0.05) between head kidney lipid and fatty acid composition (% of total) in Atlantic salmon fed diets with different ratios of ω6:ω3 and levels of EPA+DHA. TAG, PL, and ST represent triacylglycerol, phospholipids, and sterols, respectively. ΣSFA, ΣMUFA, and ΣPUFA represent total saturated, monounsaturated, and polyunsaturated fatty acids, respectively.

| Lipid class | FA | Pearson r | p-value |
| --- | --- | --- | --- |
| TAG | 16:0 | -0.36 | 0.03 |
|  | 18:0 | -0.44 | 0.01 |
|  | 18:1ω9 | 0.55 | 0.0001 |
|  | 20:4ω6 (ARA) | -0.41 | 0.01 |
|  | 22:6ω3 (DHA) | -0.45 | 0.01 |
|  | ΣSFA | -0.35 | 0.04 |
|  | ΣMUFA | 0.49 | 0.002 |
| PL | 18:2ω6 (LNA) | -0.40 | 0.01 |
|  | 20:2ω6 | -0.34 | 0.04 |
|  | 20:5ω3 (EPA) | 0.41 | 0.01 |
|  | ω6:ω3 | -0.34 | 0.04 |
| ST | 18:1ω9 | -0.70 | 0.0001 |
|  | 20:3ω6 (DGLA) | 0.43 | 0.01 |
|  | 20:4ω6 (ARA) | 0.57 | 0.0001 |
|  | 22:6ω3 (DHA) | 0.44 | 0.01 |
|  | ΣMUFA | -0.71 | 0.0001 |
|  | ΣPUFA | 0.49 | 0.002 |
|  | EPA:ARA | -0.49 | 0.002 |

^a^ Pearson correlations were calculated using individual fish (n = 6–8).
